# Reduced PRC2 function alters male germline epigenetic programming and paternal inheritance

**DOI:** 10.1186/s12915-018-0569-5

**Published:** 2018-09-20

**Authors:** Jessica M. Stringer, Samuel C. Forster, Zhipeng Qu, Lexie Prokopuk, Moira K. O’Bryan, David K. Gardner, Stefan J. White, David Adelson, Patrick S. Western

**Affiliations:** 1 Centre for Reproductive Health, Hudson Institute of Medical Research, Clayton, Victoria 3168 Australia; 2 Department of Anatomy and Developmental Biology, Ovarian Biology Laboratory, Biomedicine Discovery Institute, Monash University, Melbourne, 3168 Australia; 3 Host-Microbiota Interactions Laboratory, Wellcome Trust Sanger Institute, Hinxton, CB10 1SA UK; 4 Centre for Innate Immunity and Infectious Diseases, Hudson Institute of Medical Research, Clayton, Victoria 3168 Australia; 5 Molecular and Translational Science, Monash University, Clayton, Victoria 3168 Australia; 6 Bioinformatics and Computational Genetics, School of Biological Sciences, The University of Adelaide, Adelaide, South Australia 5005 Australia; 7 School of Biological Sciences, Monash University, Clayton, Victoria 3168 Australia; 8 School of BioSciences, University of Melbourne, Parkville, Australia; 9 Department of Human Genetics, Leiden Genome Technology Centre, Leiden University Medical Center, Leiden, the Netherlands

**Keywords:** Germline, Epigenetic reprogramming, PRC2, H3K27me3, Paternal inheritance, Fertility

## Abstract

**Background:**

Defining the mechanisms that establish and regulate the transmission of epigenetic information from parent to offspring is critical for understanding disease heredity. Currently, the molecular pathways that regulate epigenetic information in the germline and its transmission to offspring are poorly understood.

**Results:**

Here we provide evidence that Polycomb Repressive Complex 2 (PRC2) regulates paternal inheritance. Reduced PRC2 function in mice resulted in male sub-fertility and altered epigenetic and transcriptional control of retrotransposed elements in foetal male germ cells. Males with reduced PRC2 function produced offspring that over-expressed retrotransposed pseudogenes and had altered preimplantation embryo cleavage rates and cell cycle control.

**Conclusion:**

This study reveals a novel role for the histone-modifying complex, PRC2, in paternal intergenerational transmission of epigenetic effects on offspring, with important implications for understanding disease inheritance.

**Electronic supplementary material:**

The online version of this article (10.1186/s12915-018-0569-5) contains supplementary material, which is available to authorized users.

## Background

Numerous studies have investigated the inheritance of physiological effects caused by environmental impacts on the parental genome, but the underlying epigenetic mechanisms regulating such inheritance are poorly understood [1, 2]. It is well established that DNA methylation is passed through the germline (oocytes and sperm) to the following generation, where it influences gene activity, embryonic development and post-natal life [1, 3–5]. In addition, recent studies have demonstrated effects of histone demethylases on inheritance [6, 7]. For example, zygotic over-expression of the Histone 3 lysine 27 (H3K27) demethylase, *Kdm6b*, demonstrated a role for maternal H3K27 methylation in regulating DNA methylation-independent imprinting [7]. Similarly, increased levels of histone 3 lysine 4 dimethylation (H3K4me2) in developing sperm resulted in paternally transmitted effects on health and development in mice [6]. In this study, we provide evidence that epigenetic inheritance in mice is also altered by a hypomorphic mutation in *embryonic ectoderm development* (*Eed*), a gene that is essential for H3K27 trimethylation (H3K27me3).

H3K27me3 is mediated by Polycomb Repressive Complex 2 (PRC2), which is comprised of the essential protein components EED, EZH2 and SUZ12 [8]. In mice, complete loss of function of any of these components results in loss of PRC2 activity, global reduction in H3K27me3 and embryonic lethality [9–12]. While complete loss of *Eed* results in lethality at gastrulation [13], germ cell-specific deletion results in male sterility [14]. However, an *N*-ethyl-*N*-nitrosourea (ENU)-induced hypomorphic allele, *Eed*^*l7Rn51989SB*^, compromises PRC2 function and is compatible with survival, although some foetuses are lost during gestation due to defective placental development [13, 15]. *Eed*^*l7Rn5-1989SB*^ mice carry a point mutation at nucleotide 1989 that disrupts function of one of the WD repeat domains in the EED protein. This hypomorphic mutation does not abrogate the ability of EED to mediate H3K27 methylation as the *Eed*^*l7Rn5-1989SB*^ allele can rescue H3K27 methylation in ES cells lacking the *Eed* gene [16]. Moreover, despite low EED function, adult mice with the hypomorphic *Eed*^*l7Rn5-1989SB*^ mutation are fertile [17], allowing the investigation of PRC2 in epigenetic inheritance.

During embryonic development, epigenetic information is reprogrammed in the germline to ensure transmission of the correct information to the next generation. This involves extensive reorganisation of histone modifications and the removal of almost all DNA methylation from foetal germ cells [18–24]. In mice, removal of DNA methylation is initiated in migrating germ cells at around embryonic day (E)9, but is not complete until E13.5, after the germ cells have entered the developing gonads. Entry of germ cells into the gonads coincides with the removal of DNA methylation from imprinting control regions (ICRs), non-imprinted intergenic and intronic sequences and from many transposable elements (TEs), including LINE and SINE elements [18, 22–26]. During germline reprogramming, LINE and SINE elements are likely repressed by mechanisms other than DNA methylation to prevent TE expression and consequent insertional mutations [18, 26].

H3K27me3 broadly regulates developmental gene expression through its ability to repress target gene transcription. In foetal germ cells, H3K27me3 is enriched at developmental genes and on the 5′ flanking regions of some TEs, including LINE1 elements, intergenic regions, introns and imprint control regions [26–29]. Loss of function of the H3K9me3 methyltransferase SET domain Bifurcated 1 (SETDB1) in the developing male germline results in loss of DNA methylation, H3K9me3 and H3K27me3 at a subset of TEs [26]. This suggests that H3K27me3 functions with DNA methylation and H3K9me3 to co-regulate specific TEs in the germline [26]. Similarly, in cultured embryonic stem cells, H3K27me3 represses TEs in the absence of DNA methylation, establishing a functional requirement for H3K27me3 on these sequences [30].

H3K27me3 is enriched in foetal germ cells and in germ cells undergoing spermatogenesis [28, 29, 31, 32]. Moreover, H3K27me3 has been detected at developmental gene promoters in mature sperm, indicating that H3K27me3 may be transmitted to offspring and that such genes are poised for activation in the preimplantation embryo [33–36]. Another study showed retention of nucleosomes at repetitive sequences in sperm, including at LINE elements [37–39]. Together, these studies raise the possibility that PRC2 and H3K27me3 regulate TEs during germline reprogramming and may modulate epigenetic inheritance in offspring. However, whether the potential inherited effects are directly mediated by histone modifications in offspring, or involve other mechanisms such as DNA methylation or altered inheritance of RNAs is unknown.

The aim of this study was to determine whether PRC2 contributes to the regulation of paternal epigenetic inheritance in a mammalian model. Using the hypomorphic *Eed*^*l7Rn5-1989SB*^ mice, we provide evidence that PRC2 modulates H3K27me3 enrichment on TEs and represses retrotransposable LINE elements in the foetal male germline. Moreover, our data indicate that PRC2 is required in the paternal germline to regulate offspring development and repress a cohort of retrotransposed pseudogenes and related lincRNAs in offspring.

## Results

### *Eed*^*l7Rn5-1989SB*^ mice are sub-fertile and provide a model for the study of epigenetic inheritance through the paternal germline

Since the primary aim of this study was to determine the role of EED in paternal epigenetic inheritance, we first assessed survival and male fertility in our colony of *Eed*^*l7Rn5-1989SB*^ mice. While the expected proportions of *Eed*^*wt/wt*^ and *Eed*^*wt/hypo*^ offspring were produced, the proportion of *Eed*^*hypo/hypo*^ mice was significantly reduced (ratio 1:2:0.1), demonstrating that *Eed*^*l7Rn5-1989SB*^ homozygosity reduces viability (Additional file 1: Figure S1A). At E15.5 *Eed*^*wt/wt*^, *Eed*^*wt/hypo*^ and *Eed*^*hypo/hypo*^ foetuses were recovered in a 1:2:0.6 ratio (Additional file 1: Figure S1A). As few still-births or neonatal deaths were observed, we concluded that most *Eed*^*hypo/hypo*^ embryos died during the second half of gestation, consistent with previous observations [13, 17]. Despite the loss of some foetuses, these experiments confirmed the survival of *Eed*^*hypo/hypo*^ males to adulthood, allowing the study of the male germline in a background of low EED function.

**Fig. 1:**
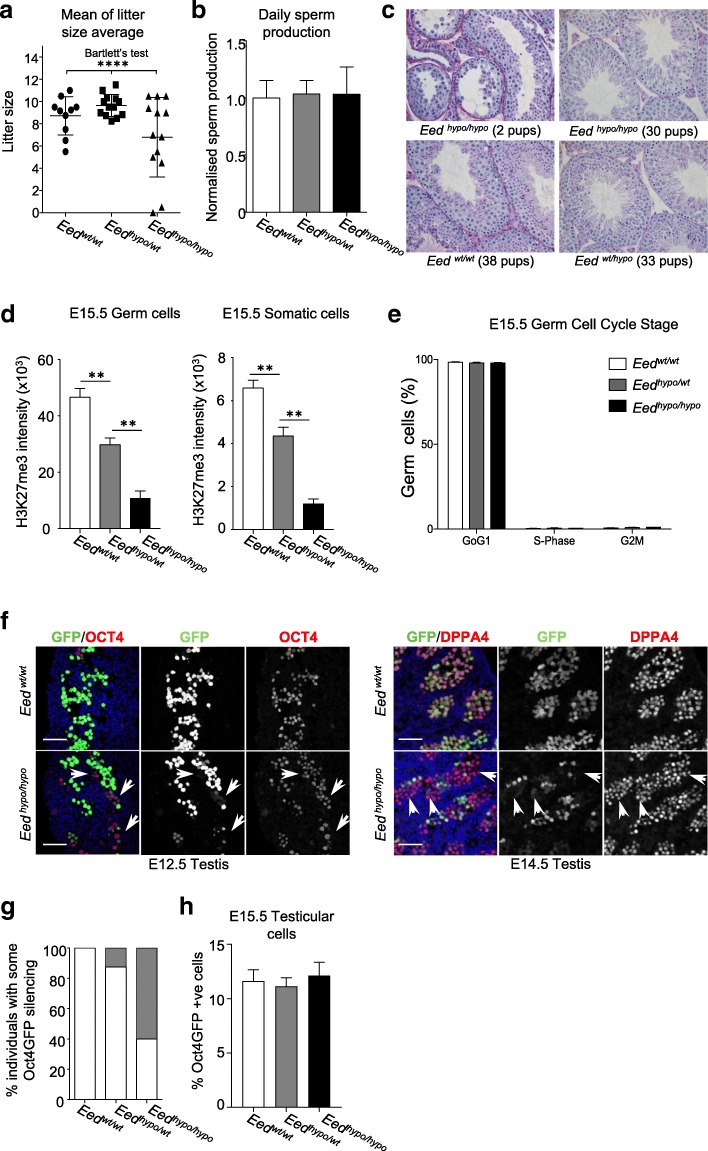
Reduced EED function resulted in male subfertility and reduced litter size. **a** Average litter size produced by wild-type female mice mated to *Eed*^*wt/wt*^ (*n* = 10), *Eed*^*hypo/wt*^ (*n* = 13) and *Eed*^*hypo/hypo*^ (*n =* 13) males mated to for two periods of 30 days. Each point represents the average of two, four or six litters per male. Average litter sizes produced by *Eed*^*hypo/hypo*^ males were more variable in size than from *Eed*^*wt/wt*^ and *Eed*^*hypo/wt*^ males (Bartlett’s test *P* = 0.0002). **b** Normalised daily sperm production of *Eed*^*wt/wt*^ (*n =* 3), *Eed*^*hypo/wt*^ (*n* = 5), and *Eed*^*hypo/hypo*^ (*n* = 6) males (mean ± SEM, one-way ANOVA; no significant differences). **c** Testis histology from sub-fertile and fertile *Eed*^*hypo/hypo*^ males (top row) compared to their *Eed*^*wt/wt*^ or *Eed*^*wt/hypo*^ siblings (bottom images). In some *Eed*^*hypo/hypo*^ males (top left panel), testis cords contained reduced numbers of germ cells with evidence of germ cell sloughing, while other cords demonstrated apparently normal spermatogenesis. In some *Eed*^*hypo/hypo*^ males, testis cords appeared normal (top right panel). The total number of pups produced from the first four females is shown in parentheses. **d**, **e** Flow cytometric analysis of H3K27me3 levels (**d**) and cell cycle stage (**e**) in germ and somatic cells of E15.5 *Eed*^*wt/wt*^, *Eed*^*wt/hypo*^ and *Eed*^*hypo/hypo*^ testes. *Eed*^*wt/wt*^ (*n* = 5 and 2), *Eed*^*hypo/wt*^ (*n* = 7 and 4) and *Eed*^*hypo/hypo*^ (*n* = 2 and 3) testes in **d** and **e** respectively. Data are mean ± SEM. **f** Immunofluorescent analysis of E12.5 and E14.5 *Eed*^*hypo/hypo*^ and *Eed*^*wt/wt*^ foetal testis sections using antibodies specific for OCT4 at E12.5 and DPPA4 at E14.5. Merged images are shown in the left panels, with greyscale images of GFP shown in the middle panels and DPPA4 in the right-hand panels. Nuclear DNA is marked by DAPI in blue. Arrows indicate germ cells that lack *Oct4*GFP expression. Scale bar 50 μm. **g** Percentage of foetuses in which *Oct4*GFP silencing was observed in some germ cells at E12.5 and E14.5 in *Eed*^*wt/wt*^ (*n* = 0/7), *Eed*^*wt/hypo*^(*n =* 1/8) and *Eed*^*hypo/hypo*^ (*n =* 6/10) animals. **h** Average percentage of cells in the total cell population that were Oct4GFP positive in pairs of E15.5 testes from *Eed*^*wt/wt*^ (*n =* 13 from 8 litters), *Eed*^*wt/hypo*^ (*n* = 31 form 17 litters) and *Eed*^*hypo/hypo*^ (*n* = 14 from 9 litters) males **(**mean ± SEM, One-way ANOVA; No significant differences)

While previous studies found that homozygous *Eed*^*l7Rn5-1989SB*^ mice produced offspring [17], the level of fertility in these mice remained unknown. We therefore completed a fertility analysis to determine whether the *Eed* hypomorphic mutation affected male germline function. Fertility was assessed in a cohort of hypomorphic *Eed*^*hypo/hypo*^ males (*n* = 13) compared to their age-matched *Eed*^*hypo/wt*^ (*n* = 13) and *Eed*^*wt/wt*^ (*n* = 10) brothers, mated to wild-type females. Females were assessed for copulatory plugs as an indication of normal mating behaviour. *Eed*^*hypo/hypo*^ males sired 6.8 ± 3.6 pups per litter compared to 9.6 ± 1.0 and 8.7 ± 1.7 pups sired by *Eed*^*hypo/wt*^ and *Eed*^*wt/wt*^ male controls, respectively. Notably, litters sired by *Eed*^*hypo/hypo*^ males were highly variable in size, resulting in a significantly increased standard deviation compared to *Eed*^*hypo/wt*^ and *Eed*^*wt/wt*^ controls (Bartlett’s test *p* = 0.0002) (Fig. 1a). *Eed*^*hypo/hypo*^ males produced no pups or small litters more frequently than *Eed*^*hypo/wt*^ and *Eed*^*wt/wt*^ males (Additional file 1: Figure S1B; chi-square *P* = 1.8E−05), indicating sub-fertility in some *Eed*^*hypo/hypo*^ males. No difference was observed in the average daily sperm count between genotypes (Fig. 1b), and there was no correlation between litter size or frequency and male age (Additional file 1: Figure S1C).

Consistent with sporadic sub-fertility, testicular morphology of *Eed*^*hypo/hypo*^ males was also variable, but consistent with fertility outcomes. In obviously sub-fertile *Eed*^*hypo/hypo*^ males, germ cells were reduced and vacuoles present in some testis cords, indicating that germ cells were lost through sloughing (Fig. 1c). By contrast, normal testicular morphology was observed in *Eed*^*hypo/hypo*^ males that produced normal litter sizes (Fig. 1c). As a cohort (*n* = 20), abnormal testicular histology was only observed in four *Eed*^*hypo/hypo*^ males, with the remainder maintaining apparently normal testes with qualitatively normal spermatogenesis and weight. Combined, these data reflect the relatively mild sub-fertility and testicular phenotypes observed in these mice.

To determine whether the *Eed* hypomorphic mutation affected testis formation, we assessed the impact of reduced EED function on H3K27me3 levels and the development of germ and somatic cells in foetal testes. E12.5 and E15.5 were examined as they represent the earliest stages of testis formation and male germline development, and the completion of PRC2-dependent reorganisation of H3K27me3 and initiation of DNA re-methylation in the paternal germline, respectively [1, 3–5, 40]. While H3K27me3 was detected in germ cells of all genotypes by immunofluorescence (Additional file 1: Figure S2A), flow cytometric assessment revealed significantly reduced global H3K27me3 levels in E15.5 germ and somatic cells in *Eed*^*hypo/wt*^ and *Eed*^*hypo/hypo*^ compared to *Eed*^*wt/wt*^ testes (Fig. 1d, Additional file 1: Figure S2B). Reduced levels of H3K27me3 were presumably due to hypomorphic function of EED, as EED, EZH2 and SUZ12 were all detected in the germ and somatic cells of *Eed*^*wt/wt*^, *Eed*^*hypo/wt*^ and *Eed*^*hypo/hypo*^ testes (Additional file 1: Figure S2A).

Although H3K27me3 levels were reduced, the percentage of Sertoli and germ cells in the gonad (Additional file 1: Figure S2C-D), the proliferation of Sertoli cells (Additional file 1: Figure S2E) and the entry of germ cells into mitotic arrest (Fig. 1e) was unaffected. Similarly, qRTPCR analyses of a range of testis development genes involved in Sertoli, germ and steroidogenic cell development in E12.5 and E15.5 foetal testes (Additional file 1: Figure S2F), and flow cytometric analysis of SOX9 and AMH (Additional file 1: Figure S2G-H) revealed no differences between *Eed*^*wt/wt*^ *Eed*^*hypo/wt*^ and *Eed*^*hypo/hypo*^ testes. Collectively, these data demonstrated that reduced EED function significantly affected male fertility and germline H3K27me3 levels. However, the majority of males were able to produce litters in which epigenetic inheritance could be effectively studied.

### Reduced EED function resulted in stochastic silencing in male foetal germ cells

To facilitate isolation of germ cells, our *Eed* hypomorphic mice carried a randomly integrated *Oct4*GFP transgene that is robustly transcribed in all foetal germ cells until birth, but remains silent in somatic cells [41, 42]. Initially, to confirm the veracity of the Oct4GFP transgene in this model, we used immunofluorescence to examine OCT4, DPPA4 and MVH in germ cells of E12.5 *Eed*^*wt/wt*^ *Eed*^*hypo/wt*^ and *Eed*^*hypo/hypo*^ testes (Additional file 1: Figure S2I). Although OCT4, DPPA4 and MVH were detected in all germ cells, we observed silencing of *Oct4*GFP in some small patches of germ cells in some germ cells at E12.5 and E14.5 (Fig. 1f). However, this was not fully penetrant, as it affected ~ 60% of *Eed*^*hypo/hypo*^ and ~ 10% of *Eed*^*wt/hypo*^ individuals, and silencing was only evident in small numbers of germ cells (Fig. 1g). Indeed, analysis of FACS data revealed that there was no difference in the proportion of *Oct4*GFP-positive cells obtained from E15.5 foetal testes of *Eed*^*hypo/hypo*^, *Eed*^*wt/hypo*^ and *Eed*^*wt/wt*^ animals (Fig. 1h). This was consistent with similar numbers of MVH-positive germ cells in the testes of E15.5 *Eed*^*hypo/hypo*^, *Eed*^*wt/hypo*^ and *Eed*^*wt/wt*^ animals (Additional file 1: Figure S2C). Combined, these data demonstrated that although normal numbers of foetal germ cells were present in *Eed*^*hypo/hypo*^ testes, occasional stochastic silencing of *Oct4*GFP occurred in germ cells of males with reduced EED function. As transgene silencing has been observed in other epigenetic models [43–45], we proposed that the stochastic Oct4GFP silencing in *Eed*^*hypo/hypo*^ germ cells was indicative of an altered epigenetic state in the germline of *Eed* hypomorphic males.

### H3K27me3 is required to repress LINE elements in male foetal germ cells

To determine whether epigenetic state was disrupted in E15.5 male foetal germ cells we used ChIP-seq analysis to assess H3K27me3 enrichment in germ cells isolated from each of four single *Eed*^*hypo/hypo*^ male embryos and four *Eed*^*wt/wt*^ male embryos. ChIP-seq yielded averages of 20,139,219 and 20,195,969 reads from the *Eed*^*hypo/hypo*^ and *Eed*^*wt/wt*^ samples, respectively, of which 97.2–97.9% were alignable to the mm10 reference genome using bowtie2 (Additional file 1: Figure S3). HOMER analysis using a search region size of 1100 bp identified 60,933 and 55,453 H3K27me3 peaks in the *Eed*^*hypo/hypo*^ and *Eed*^*wt/wt*^ samples, respectively (Additional file 2: Table S1 and Additional file 3: Table S2)*.* Importantly, comparison of our data to three similar datasets demonstrated significant overlap at known PRC2 targets (Additional file 1: Figure S3G, Additional file 4: Table S3), demonstrating high specificity of the ChIP analysis. In addition, visualisation of normalised read counts in the *Eed*^*hypo/hypo*^ and *Eed*^*wt/wt*^ germ cell samples demonstrated clear H3K27me3 enrichment in the 5-prime regions of PRC2 target genes and no enrichment on a constitutively expressed gene *Sdha*, demonstrating sensitivity of the assay (Additional file 1: Figure S3H).

**Fig. 2:**
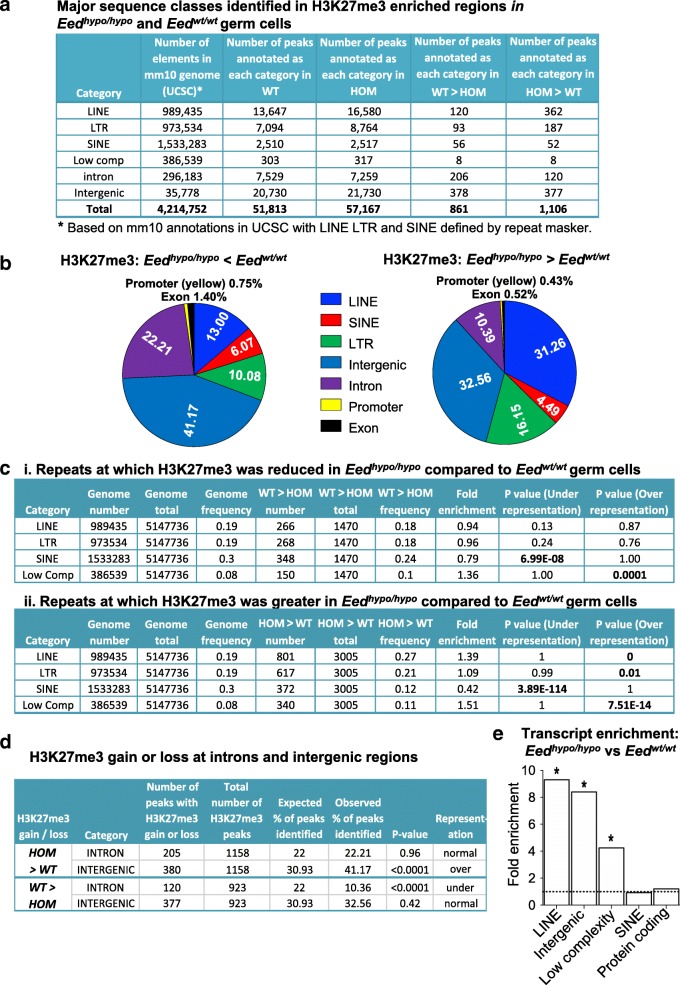
Reduced EED leads to epigenetic dysregulation of transposable elements in the paternal germline**. a** Major sequence classes identified with H3K27me3 enrichment identified by ChIP-seq in FACS purified E15.5 *Eed*^*wt/wt*^ (*n* = 4) and *Eed*^*hypo/hypo*^ (*n =* 4) germ cells. **b** Graphical summary of regions with loss or gain of H3K27me3 in *Eed*^*hypo/hypo*^ germ cells (*n =* 4) compared to *Eed*^*wt/wt*^ germ cells (*n =* 4). **c** Hypergeometric analysis of the expected and observed representation of sequences on which H3K27me3 was reduced (i) or increased (ii) in *Eed*^*hypo/hypo*^ (*n =* 4) compared to *Eed*^*wt/wt*^ (*n =* 4) male E15.5 germ cells, grouped by annotation. *P* values for over- and under-representation of each sequence category are shown, with *P* < 0.05 considered significant. **d** Chi-square analysis of the expected and observed representation of sequences on which H3K27me3 was increased (HOM > WT) or reduced (WT > HOM) in *Eed*^*hypo/hypo*^ (*n =* 4) compared to *Eed*^*wt/wt*^ (*n =* 4) male E15.5 germ cells, grouped by annotation. Intergenic sequences were significantly over-represented in sequences gaining H3K27me3, while intronic sequences were significantly under-represented in sequences losing H3K27me3 in *Eed*^*hypo/hypo*^ germ cells. **e** Fold enrichment of transcripts from LINE, intergenic, low complexity, SINE and protein-coding genes in germ cells from E15.5 *Eed*^*hypo/hypo*^ (*n =* 4) and *Eed*^*wt/wt*^ (*n =* 4) foetuses. *P < 0.05 (Fisher exact test). Dotted line illustrates no enrichment relative to random distribution

In addition, a high proportion of peaks were identified at repeat elements including LINE, LTR and SINE elements in both *Eed*^*hypo/hypo*^ and *Eed*^*wt/wt*^ germ cell samples (Fig. 2a). We used hypergeometric testing to determine whether the expected number of repeats was represented for each repeat category in the ChIP-seq data for *Eed*^*wt/wt*^ and *Eed*^*hypo/hypo*^ germ cells. In both *Eed*^*hypo/hypo*^ and *Eed*^*wt/wt*^ samples, SINE elements were substantially under-represented (fold enrichment = 1.31 and 1.22, respectively; *p*~ 0) in H3K27me3-enriched peaks, but LINE elements were substantially over-represented (fold enrichment = 0.51 and 0.61, respectively; *P*~ 0; Additional file 5: Table S4), suggesting that LINE elements were preferentially captured in the ChIP seq assay, but SINE elements were not (Additional file 5: Table S4). LTRs were represented at expected ratios in *Eed*^*hypo/hypo*^ and *Eed*^*wt/wt*^ samples (fold enrichment = 1.04 and 1.0, respectively; Additional file 5: Table S4). Together, these data indicated strong enrichment of H3K27me3 not only at PRC2 target loci, but also at some repeat sequences, most notably LINE elements.

To investigate locus-specific variation in H3K27me3, we used HOMER to identify specific sequences with differential H3K27me3 enrichment by comparing *Eed*^*hypo/hypo*^ samples to *Eed*^*wt/wt*^ samples (Additional file 1: Figure S3I-J). As H3K27me3 has previously been implicated in repression of both coding and non-coding sequences in foetal male germ cells [26, 28], we included repetitive sequences in our analyses. Samples were compared using *Eed*^*wt/wt*^ as the baseline target and searching for regions with a cumulative Poisson *P* value less than 0.0001 (sequencing-depth dependent) and ≥ 2-fold reduction in H3K27me3 precipitated sequences. This revealed 923 regions with ≥ 2-fold reduction in H3K27me3 in *Eed*^*hypo/hypo*^ compared *Eed*^*wt/wt*^ germ cells (i.e. WT > HOM). The reciprocal comparison using *Eed*^*hypo/hypo*^ samples as baseline and *Eed*^*wt/wt*^ samples as target identified 1,158 regions with ≥ 2-fold increased H3K27me3 in *Eed*^*hypo/hypo*^ germ cells (i.e. HOM > WT). Only 58 of these regions associated with coding genes (35 decreased and 23 increased, 2.84% of all differential peaks), while 1,951 LINE, LTR, SINE, intergenic and intronic sequences were identified with significantly different (≥ 2-fold) levels of H3K27me3 (Fig. 2a; Additional file 6: Table S5 and Additional file 7: Table S6). These included 120 LINE, 93 LTR, 56 SINE, 378 intergenic and 206 intronic with decreased (Fig. 2a) and 362 LINE, 187 LTR, 52 SINE, 377 intergenic and 120 intronic with increased (Fig. 2a) H3K27me3 in *Eed*^*hypo/hypo*^ compared to *Eed*^*wt/wt*^ germ cells, representing > 97% of all differential ChIP peaks (Fig. 2b). Using a more stringent analysis employing edgeR, with a false discovery rate cut off of *P* < 0.05, we identified 7 refGene annotated LINE1 loci with significantly fewer reads mapped in *Eed*^*hypo/hypo*^ compared to the *Eed*^*wt/wt*^ samples (Additional file 8: Table S7), but no LINE1 elements with significantly more reads mapped in *Eed*^*hypo/hypo*^ compared to the *Eed*^*wt/wt*^ samples. Together, these data indicated that subtle differences in H3K27me3 regulation occurred predominantly at repetitive sequences, introns and intergenic regions in *Eed*^*hypo/hypo*^ germ cells.

We next examined the representation of each sequence category with differential H3K27me3 enrichment in the ChIP-seq data relative to the expected representation of annotated sequence classes across the genome. For repeats, hypergeometric testing was used to analyse the representation for each repeat category gaining or losing H3K27me3 in *Eed*^*wt/wt*^ and *Eed*^*hypo/hypo*^ germ cells relative to the total number of repeats in the genome. For example, LINE elements occupy 19% of all genome repeats (Fig. 2c (i)), and 18% of repeats were identified with reduced H3K27me3 in *Eed*^*hypo/hypo*^ compared to *Eed*^*wt/wt*^ germ cells which were LINE elements (Fig. 2c (i)). In contrast, 27% of repeat sequences were identified with increased H3K27me3 in *Eed*^*hypo/hypo*^ compared to *Eed*^*wt/wt*^ germ cells which were LINE elements, a significantly higher proportion than the expected 19% (Fig. 2c (ii)). Thus, LINE elements with increased H3K27me3 were significantly over-represented in *Eed*^*hypo/hypo*^ germ cells (Fig. 2c; fold enrichment 1.39, *P* ~ 0), but LINE elements with reduced H3K27me3 were detected at the expected frequency. LTRs were very moderately over-represented in peaks with increased H3K27me3 in *Eed*^*hypo/hypo*^ germ cells (Fig. 2c; observed 21%, expected 19%; enrichment ratio 1.09; *P* = 0.01). In contrast, SINE elements were under-represented in repeats with either reduced or increased H3K27me3, a result that was consistent with overall under-representation of SINE elements in both the *Eed*^*hypo/hypo*^ and *Eed*^*wt/wt*^ germ cell H3K27me3 ChIPseq datasets (Fig. 2c). Although very few low complexity repeats had altered H3K27me3 in the ChIPseq dataset, these sequences were over-represented in both H3K27me3 gain and loss categories (i.e. some low complexity sequences gained H3K27me3, while others lost H3K27me3).

For non-repetitive genomic sequences (e.g. intergenic, intronic and promoters), we determined whether the expected percentage of peaks was represented for each sequence category in the ChIPseq data relative to the percentage of the total genome occupied by each sequence category. For example, intergenic sequences occupy 30.93% of the genome, but were represented at normal frequency in peaks with decreased (32.96%, *P* = 0.42, chi-square analysis) but were over-represented in peaks with increased H3K27me3 (41.17%, *P* < 0.0001; Fig. 2d). In contrast, intronic sequences were under-represented in peaks losing H3K27me3 in *Eed*^*hypo/hypo*^ germ cells but were normally represented in peaks gaining H3K27me3 (Fig. 2d). Promoters, exons, 5′UTRs, 3′UTRs, small RNAs, tRNAs, rRNAs and CpG islands were not significantly over- or under-represented in peaks with either increased or decreased H3K27me3 in *Eed*^*hypo/hypo*^ compared to *Eed*^*wt/wt*^ germ cells. Together, these data indicated that H3K27me3 was redistributed throughout the genome of *Eed*^*hypo/hypo*^ compared to *Eed*^*wt/wt*^ germ cells. Although this apparently resulted in increased H3K27me3 at some intergenic and repeat sequences, significant numbers of LINE, LTR, SINE elements, intergenic and intronic sequences were detected with reduced H3K27me3.

Since H3K27me3 is a repressive modification, reduced H3K27me3 may result in increased transcription from the underlying sequence. To determine whether this was the case, RNA sequencing (RNA-seq) was performed to an average depth of > 20 million reads per sample on FACS purified E15.5 male foetal germ cells using four independent *Eed*^*hypo/hypo*^ and four *Eed*^*wt/wt*^ samples (Additional file 1: Figure S4A). Comparison of 1000 genes that were not differentially expressed indicated a high level of technical consistency between the sample sets. (Additional file 1: Figure S4B). Similarly, principal component analysis revealed strong correlation between the RNA-seq sample sets generated from *Eed*^*wt/wt*^ germ cells (Additional file 1: Figure S4C), although, notably, there appeared to be greater variation between samples from *Eed*^*hypo/hypo*^ germ cells. This was reminiscent of the observed stochastic variation in *Oct4GFP* expression in E15.5 germ cells in *Eed*^*hypo/hypo*^ mice, but not in *Eed*^*wt/wt*^ mice. Consistent with the lack of differences in H3K27me3 enrichment at protein-coding genes, we observed no differences in expression of protein-coding genes in *Eed*^*hypo/hypo*^ compared to *Eed*^*wt/wt*^ germ cells using a significance limit of *P* < 0.01 with Benjamini-Hochberg false detection correction. However, analysis of repetitive sequences annotated using HOMER, including TEs, revealed significant enrichment of RNA-seq reads mapping to annotated LINE elements (*P* = 0.033, Fisher Exact Test, Benjamini-Hochberg false detection correction), intergenic (*P* = 0.0364, Fisher Exact Test, Benjamini-Hochberg false detection correction) and low complexity sequences (*P* = 0.025, Fisher Exact Test, Benjamini-Hochberg false detection correction) in *Eed*^*hypo/hypo*^ germ cells, although reads mapping to SINE sequences and protein coding sequences remained unchanged (Fig. 2e). Although increased transcription of LINE elements was observed as a class in E15.5 *Eed*^*hypo/hypo*^ germ cells compared to controls, we could not identify specific LINE sequences that were consistently dysregulated. Given the stochastic variation observed in *Oct4*GFP expression (Fig. 1f, g), and the increased variation between *Eed*^*hypo/hypo*^ germ cell samples in the RNA-seq data (Additional file 1: Figure S4C), a plausible explanation for this is that a specific LINE element may be affected at one loci in one cell, but not affected in another cell,  resulting in variation across the whole cell population.

Despite this caveat, these combined RNA-seq and ChIP-seq data demonstrate that H3K27me3 was substantially redistributed on LINE, SINE and LTR elements and on intergenic and intronic regions, but not on protein-coding genes in germ cells of *Eed*^*hypo/hypo*^ mice. Moreover, although only a subset of retrotransposed LINE elements showed reduced H3K27me3, this class of repeats showed almost 10-fold increased global expression in *Eed*^*hypo/hypo*^ compared to *Eed*^*wt/wt*^ germ cells.

### Paternal PRC2 regulates retrotransposed pseudogene silencing in offspring

**Fig. 3:**
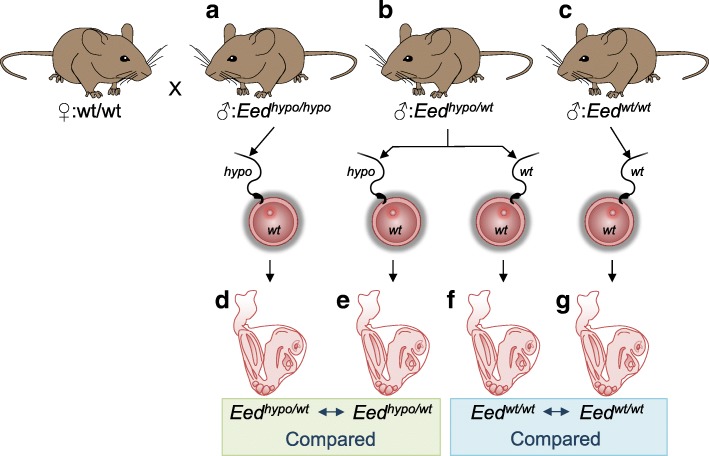
Breeding and experimental plan to assess epigenetic inheritance. Wild-type (*wt/wt*) females that had never been exposed to the *Eed* hypomorphic mutation were mated to either **a** homozygous (*Eed*^*hypo/hypo*^), **b** heterozygous (*Eed*^*hypo/wt*^) males (*n =* 3 littermate pairs for each genotype) or **c** wild-type (*Eed*^*wt/wt*^, *n =* 2) males. Sperm in heterozygous and wild-type males develop with at least one fully functional copy of *Eed* (**b** and **c**), while sperm in homozygous hypomorphic males (**a**) develop without normally functioning *Eed* and are expected to contain altered epigenetic patterning. All oocytes are wild-type. **d–g** Stage- and size-matched E8.5-day embryos were collected for transcriptional analysis. Comparisons between heterozygous embryos (**d** and **e**) and between wild-type embryos (**f** and **g**) identified genes that were misregulated due to non-genetic differences inherited from the sperm

The altered H3K27me3 enrichment, increased transcription of retrotransposed elements in foetal male germ cells and the stochastic silencing of the *Oct4*GFP transgene was strongly suggestive of epigenetic dysregulation in the developing male germ cells. We therefore established a model to investigate whether PRC2-mediated epigenetic dysregulation in the paternal germline might lead to inherited defects in offspring. We hypothesised that sperm developing from diploid germ cells with reduced PRC2 function (*Eed*^*hypo/hypo*^) would have disrupted epigenetic patterning and produce offspring with altered gene expression profiles. In this model, *Eed*^*hypo/hypo*^ males produce *Eed*^*hypo*^ sperm that develop in the absence of normal EED, while *Eed*^*hypo/wt*^ males produce *Eed*^*hypo*^ sperm that develop in the presence of a normal functioning *Eed* allele. Based on this differential EED content, mating of these males with normal wild-type females would allow the detection of paternally transmitted epigenetic effects in the absence of any confounding maternal contributions. Critically, comparison of offspring with the same *Eed*^*hypo/wt*^ genotype produced from *Eed*^*hypo/hypo*^ and *Eed*^*hypo/wt*^ fathers would reveal differences in gene expression due to altered epigenetic patterning in the sperm (Fig. 3). Similarly, epigenetic differences could also exist in sperm produced by *Eed*^*wt/wt*^ and *Eed*^*hypo/wt*^ males due to reduced EED function in the germline of *Eed*^*hypo/wt*^ males.

**Fig. 4:**
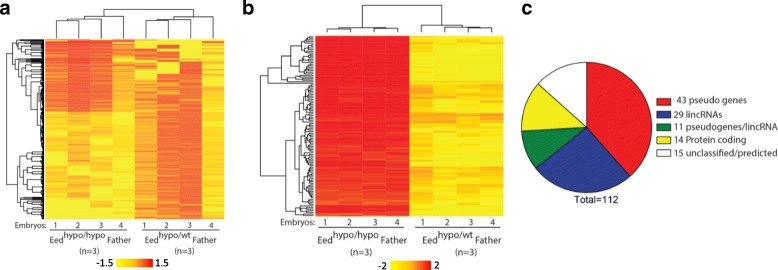
Global transcription is altered in offspring of *Eed*^*hypo/hypo*^ males. **a** Heatmap of 1986 differentially expressed transcripts (*P* < 0.01, ≥ 1.5-fold change; Benjamini-Hochberg false detection correction) detected using RNA-seq. Each column represents an RNA sample from an individual heterozygous embryo sired by either a homozygous (*Eed*^*hypo/hypo*^) or heterozygous (*Eed*^*hypo/wt*^) male. Three different *Eed* littermate pairs (i.e. *Eed*^*hypo/hypo*^ and *Eed*^*hypo/wt*^ brothers) were used to generate progeny for each genotype. **b** Heat map of 112 differently expressed genes detected using both Agilent 8x60K arrays and RNA-seq (*P* < 0.01; ≥ 2-fold change; Benjamini-Hochberg false detection correction for arrays and RNAseq). Each column (1–4) represents an RNA sample from an individual heterozygous embryo sired by either an *Eed*^*hypo/hypo*^ or an *Eed*^*hypo/wt*^ male. **c** Pie chart illustrating functional classification of differentially regulated genes detected using micro-array and RNA-seq. Processed retrotransposed pseudogenes (*P* < 4 × 10^− 7^, Fisher exact test) and lincRNAs (*P* < 0.05, Fisher exact test) were significantly enriched in genes upregulated in *Eed*^*hypo/wt*^ progeny of *Eed*^*hypo/hypo*^ compared to *Eed*^*hypo/wt*^ males

To test this model, independent *Eed*^*hypo/hypo*^, *Eed*^*hypo/wt*^ and *Eed*^*wt/wt*^ males were mated with *Eed*^*wt/wt*^ females of the same background that had never been exposed to the *Eed* mutation (Fig. 3). *Eed*^*hypo/hypo*^ and *Eed*^*hypo/wt*^ brothers produced from three independent mating pairs were used to sire *Eed*^*hypo/wt*^ embryos. Precisely staged and size matched E8.5 heterozygous and wild-type progeny were collected from each cross and photographed. Whole genome gene expression profiles of heterozygous progeny from *Eed*^*hypo/hypo*^ and *Eed*^*hypo/wt*^ males were determined using RNA-seq at a depth of ~ 40 million reads per sample (*n* = 4 offspring from each group; 40.75±7 and 51.3±13 million reads per sample for *Eed*^*hypo/hypo*^ or three *Eed*^*hypo/wt*^ sires, respectively; Fig. 4a).

Comparison of gene expression patterns in E8.5 *Eed*^*hypo/wt*^ embryos sired by three *Eed*^*hypo/hypo*^ or three *Eed*^*hypo/wt*^ sibling males (Fig. 4a) identified 1986 differentially expressed transcripts, representing at least 1851 genetically distinct genes separated by more than 5000 bp (*P* < 0.01, Benjamini-Hochberg false detection correction) (Fig. 4b).

Of these genes, 779 exhibited greater than 1.5-fold change in expression between *Eed*^*hypo/wt*^ heterozygous offspring produced by *Eed*^*hypo/hypo*^ males and *Eed*^*hypo/wt*^ males. These data demonstrated that there were transcriptional differences between *Eed*^*hypo/wt*^ offspring that result from altered PRC2 function in the paternal germline.

To confirm these changes, Agilent 8x60K arrays were used to perform a technically independent assessment of gene expression in the same E8.5 embryos. A total of 234 differentially expressed transcripts, representing 128 distinct genes, were identified between the offspring from *Eed*^*hypo/hypo*^ and *Eed*^*hypo/wt*^ fathers (*P* < 0.01; ≥ 2-fold change; Benjamini-Hochberg false detection correction) (Fig. 4c). Of these 128 genes, 112 were also identified as differentially regulated in the RNA-seq analysis (Additional file 9: Table S8). Moreover, the direction of change (up- or downregulation) for the transcripts identified by array correlated with the RNA-seq analyses (*R*^2^ = 0.87) (Additional file 1: Figure S5A). Mapping analyses revealed localization of these genes across all autosomes and the X chromosome (Additional file 1: Figure S5B). By contrast, comparison of *Eed*^*wt/wt*^ embryos produced by *Eed*^*hypo/wt*^ and *Eed*^*wt/wt*^ fathers (*n* = 4 each) using Agilent 8x60K arrays did not identify any significant differentially expressed genes (Additional file 1: Figure S5C; cut-off: > 2-fold change and *P* < 0.01 with Benjamini-Hochberg false detection correction). This indicates that having at least one wild-type *Eed* allele is sufficient to support normal paternal epigenetic inheritance.

Gene ontology analysis of the 112 differentially expressed genes identified by the array and RNAseq analyses revealed significant enrichment for processed retrotransposed pseudogenes (*P* < 4 × 10^− 7^, Fisher Exact test) and lincRNAs (*P* < 0.05, Fisher Exact test) (Fig. 4d). Further examination using Retrofinder in UCSC (Retroposed Genes V6, UCSC) identified 54 expressed retrotransposed sequences (pseudogenes and pseudogenes/lincRNA; Fig. 4d). Typically, multiple independent copies of the same parent gene were identified, indicating that these pseudogenes are commonly regulated. In addition, GeneSpring analysis classified 40 probes as lincRNAs, 11 of which were also classified as retrotransposed pseudogenes, consistent with the established ability of pseudogenes to produce noncoding RNAs [46]. All of these retrotransposed genes and lincRNAs were upregulated in progeny of *Eed*^*hypo/hypo*^ males compared to progeny of *Eed*^*hypo/wt*^ males (Fig. 4c), suggesting a primary role for paternal EED in silencing these sequences in the offspring. With the exception of retrotransposed pseudogenes, no differences were detected in expression of other repetitive sequences, including LINE elements.

### Paternal PRC2 alters preimplantation cleavage rates and cell cycle gene expression in offspring

**Fig. 5:**
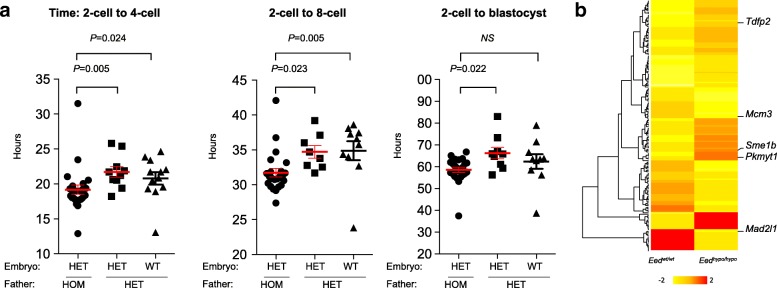
Preimplantation embryonic cleavage is advanced in offspring of *Eed*^*hypo/hypo*^ males. **a** Timing of two- to four-cell, two- to eight-cell and two-cell-blastocyst milestones in heterozygous (*Eed*^*hypo/wt*^, HET, *n =* 23) offspring sired by homozygous (*Eed*^*hypo/hypo*^, HOM) males compared to heterozygous (*Eed*^*hypo/wt*^, HET, *n* = 10) and wild-type (*Eed*^*wt/wt*^, WT *n* = 12) offspring sired by heterozygous (*Eed*^*hypo/wt*^) males mated to wild-type females (outlined in Fig. 4). Statistics: Kruskall-Wallis test with Dunn’s multiple comparisons. **b** Heat map of the top differentially expressed genes (*P* < 0.01; ≥ 2-fold change; Benjamini-Hochberg false detection correction) including *Mad2l1*, *Tdfp2*, *Mcm3*, *Pkmyt1* and *Sme1b*, which were identified as members of the KEGG cell cycle pathway using genes set enrichment analysis and the MSigDB collections

To further investigate the role of paternal germline EED function in embryonic development, we analysed preimplantation development. Zygotes produced by *Eed*^*hypo/hypo*^ and *Eed*^*hypo/wt*^ males were cultured to blastocyst stage and their development documented using automated time-lapse photography of individual embryos. Embryos were imaged every 5 min facilitating measurement of cleavage rates and embryo development to blastocyst stage. Heterozygous embryos (*n* = 24) sired by *Eed*^*hypo/hypo*^ males underwent 2–4 cell cleavage ~ 3 h earlier than heterozygous (*n* = 10, *P* = 0.0054) or wild-type embryos sired by *Eed*^*hypo/wt*^ males (*n* = 12, *P* = 0.0240; Fig. 5a). Consistent with this, time to develop from two-cell to eight-cell embryos was reduced compared to heterozygous and wild-type embryos produced by *Eed*^*hypo/wt*^ males. Time from two-cell to blastocyst was also reduced in heterozygous embryos produced by *Eed*^*hypo/hypo*^ males but was not significantly different from wild-type embryos produced by *Eed*^*hypo/wt*^ males (Fig. 5a). Collectively, these data show that preimplantation embryos from *Eed*^*hypo/hypo*^ males exhibit impaired development.

Consistent with this, RNA-seq analysis of eight-cell embryos produced by *Eed*^*hypo/hypo*^ and *Eed*^*wt/wt*^ males (*n* = 5 pools of ~ 10 embryos/sample; > 20 million reads per sample; Fig. 5b, Additional file 1: Figure S4D-F) revealed 157 transcripts with > 2-fold increased expression and 109 transcripts with decreased expression in the offspring of *Eed*^*hypo/hypo*^ males (*P* < 0.01, Benjamini-Hochberg false detection correction) (Fig. 5b; Additional file 10: Table S9 and Additional file 11: Table S10). Examination of these differentially expressed genes using GSEA identified KEGG_Cell_Cycle as the only significantly enriched pathway affected in the preimplantation progeny of *Eed*^*hypo/hypo*^ and *Eed*^*wt/wt*^ males (*q* = 0.00479). Included in this list were five genes, three of which regulate DNA replication and cell cycle progression [*Mad2l1* (11.8 fold down), *Tdfp2* and *Mcm3* (2.2-fold down)] and two that regulate meiotic progression in oocytes [*Pkmyt1* (4.7-fold up) and *Sme1b* (3.8-fold up)] (Fig. 5b, Additional file 10: Table S9 and Additional file 11: Table S10). In addition, six of the top 12 most highly upregulated genes in eight-cell embryos produced by *Eed*^*hypo/hypo*^ males were retrotransposed pseudogenes. However, analysis using HOMER revealed no differences in the expression of LINE elements or other repetitive sequences.

To determine the impacts of depleting paternal EED on peri-natal development, post-natal day (PND) 5 offspring of *Eed*^*hypo/hypo*^ and *Eed*^*hypo/wt*^ males were weighed, measured and fixed for histopathological analyses. PND5 pups produced by *Eed*^*hypo/hypo*^ and *Eed*^*hypo/wt*^ males (*n* = 2, for each genotype, each crossed with three wild-type females) were not significantly different in weight, crown-rump or nose-rump lengths (6–7 litters for each genotype, *n* = 56 and 48, respectively; litter sizes 7–9 pups, with one litter of 6 and 1 litter of 10) (Additional file 1: Figure S6). In addition, histopathological analyses examining 41 tissues were performed on PND5 male heterozygous offspring sired by three sets of sibling *Eed*^*hypo/hypo*^ (*n* = 7 offspring) and *Eed*^*hypo/wt*^ males (*n* = 6 offspring) and identified no obvious phenotypic differences.

Together, these data demonstrated that males lacking EED in the paternal germline produce offspring with altered transcriptional control of retrotransposed pseudogenes and lincRNAs. Moreover, preimplantation development was altered in these offspring, with more rapid cleavage and dysregulated control of cell cycle. Since retrotransposed sequences were also epigenetically dysregulated and over-expressed in the paternal germline, it is likely that reduced function of PRC2 in the paternal germline explains the developmental differences inherited in the offspring of *Eed*^*hypo/hypo*^ males.

## Discussion

With the exception of DNA methylation, establishment of epigenetic information in the germline and its inheritance in the following generation is poorly understood. Recent studies in mice and humans have demonstrated differential enrichment of H3K27me3 at retained nucleosomes in sperm, raising the possibility that PRC2 establishes heritable epigenetic information that significantly affects paternal offspring [34–36]. Here we identify epigenetic and transcriptional changes in the paternal germline of *Eed*^*hypo/hypo*^ males during a key period of paternal epigenetic programming. Moreover, offspring produced by *Eed*^*hypo/hypo*^ males were significantly different from offspring produced by *Eed*^*hypo/wt*^ controls at developmental and molecular levels. Since these offspring were all heterozygous for the *Eed* mutation, but were derived from sperm that developed with or without normal EED function, these observations provide prima facie evidence that PRC2 mediates epigenetic effects in the paternal germline that alter transcriptional and developmental outcomes in offspring. Consistent with roles for PRC2 in regulating intergenerational inheritance in *Drosophila*, *C. elegans* and *Xenopus* [47–51], our data support a role for PRC2 in regulating epigenetic inheritance in mammals. Moreover, a previous study demonstrated that altered function of the H3K4me3 demethylase in sperm can mediate paternally transmitted transgenerational epigenetic inheritance in mice [6]. Together, these studies strongly indicate that epigenetic inheritance is influenced by histone-modifying enzymes in mammals.

Surprisingly, although survival and male fertility were compromised in *Eed* hypomorphic animals, low EED function did not significantly alter expression patterns of protein-coding genes in developing male foetal germ cells. Consistent with this, H3K27me3 was not depleted on protein-coding genes in *Eed*^*hypo/hypo*^ male foetal germ cells, perhaps explaining why developmental gene expression remained unaltered. Similarly, in a related study we reduced global H3K27me3 levels by 80% in male foetal germ cells using the anti-EZH2 drug, GSK126, to treat gonads cultured from E12.5 to E15.5, but no significant changes in transcription of coding genes were detected using expression arrays [40]. Combined, these studies indicate that despite enrichment of H3K27me3 on many developmental genes that are not expressed in germ cells [28], these genes appear to resist upregulation when EED or EZH2 function is compromised and/or H3K27me3 levels are reduced.

Although H3K27me3 enrichment and transcription of coding genes was unaffected in foetal germ cells of *Eed*^*hypo/hypo*^ males, H3K27me3 was reduced on a substantial number of LINE, SINE and LTR elements. Moreover, transcription of LINE elements was significantly increased as a class in *Eed*^*hypo/hypo*^ germ cells, although it was not possible to identify individual TE sequences that were transcriptionally altered suggesting that variation in expression may occur at different LINE element loci on a cell to cell basis. Consistent with this, we observed silencing of the *Oct4*GFP transgene in occasional patches of germ cells in 60% of male *Eed*^*hypo/hypo*^ foetuses, indicating that activity of this transgene is subject to EED-sensitive stochastic cell to cell variation in male germ cells. Combining these observations, we propose that loss in H3K27me3 enrichment across LINE elements in the male germ cells in *Eed*^*hypo/hypo*^ mice leads to derepression of LINE elements, but this occurs in a stochastic pattern in individual cells. The impact of this across the cell population was manifest in significantly increased expression of LINE elements as a class across the cell population. Similar stochastic variation has previously been demonstrated for epigenetic regulatory mechanisms [52] and may be more pronounced in the *Eed*^*hypo/hypo*^ model than in global or tissue-specific complete loss of function (e.g. *Eed* knock out) models in which H3K27me3 is completely removed.

Together, our data highlight significant H3K27me3 enrichment on LINE, SINE and LTR elements in foetal germ cells during the period when DNA methylation begins to be re-established, supporting a role for PRC2 in germline epigenetic programming. In addition, we identified a cohort of retrotransposed pseudogenes that were derepressed in E8.5 progeny of males that had reduced function of EED in the paternal germline. Although less obvious than in the E8.5 progeny, six of the top 12 upregulated sequences in eight-cell progeny of *Eed*^*hypo/hypo*^ males were also pseudogenes. Combined, these observations provide evidence that PRC2 contributes to H3K27me3-mediated repression of LINE elements during epigenetic reprogramming in the paternal germline.

Transposable elements constitute around 45% of the genome in mammals. Some of these elements retain potential transpositional activity and must be silenced to prevent their activation and random integration in the genome [53, 54]. LINE elements encompass a group of non-LTR retrotransposons which make up around 20% of the human genome and are common to many eukaryotes [55–57]. A subset of LINE elements still retains the ability for activity and random mutagenesis; hence, strict epigenetic silencing of these sequences is vital for genome integrity [58]. Retrotransposed pseudogenes and retrotransposable elements are created by reverse transcription of processed or unprocessed mRNAs, followed by integration of these sequences back into the genome. These copies are typically imperfect in that they differ from the parent gene and accumulate mutations over time [46, 54, 59]. Our data indicate that PRC2/H3K27me3 makes an important contribution to silencing these classes of retrotransposable sequences, both in the developing germline and in the paternal progeny. While repressing retrotransposed elements is essential to prevent their mobilisation and random integration into the genome, the requirement for repressing processed pseudogenes is perhaps less obvious as they typically lack their own promoter and the ability to independently retrotranspose. However, transcribed pseudogenes can produce biologically active noncoding RNAs or proteins that have the capacity to alter cell development and function in the host organism [46, 59]. Silencing or correct transcriptional regulation of retroduplicated sequences is therefore likely to be important to preserve genome function and correct biological processes.

Several lines of evidence indicate that histone modifications are important for regulating repetitive sequence in the paternal germline. Nucleosomes are retained in repetitive sequences in sperm [37], H3K9me3 and H3K27me3 mark LTRs and LINE elements in the foetal germline [26, 29, 30], and the H3K9me3 methylase, SETDB1, is required for repression of a number retroviral elements, including some, but not all, LINE elements [26]. SETDB1 is also required to regulate inherited effects, apparently mediated through DNA methylation [60]. Moreover, LINE elements play a role in pseudogene retrotransposition [61]. Together, these observations indicate a functional link between H3K27me3 in the paternal germline and deregulation of retrotransposed pseudogenes in the offspring of PRC2 mutant males, although the mechanism through which this operates remains obscure. However, we cannot exclude the possibility that the effects mediated through PRC2 and H3K27me3 are indirect, involving other mechanisms such as altered DNA methylation or inheritance of RNAs that mediate effects in offspring.

*Eed*^*hypo/hypo*^ males produced heterozygous offspring that progressed through the two- to four-cell cleavage stage significantly earlier than heterozygous controls or wild-type offspring sired by *Eed*^*hypo/wt*^ males. Consistent with this, cell cycle genes were dysregulated in eight-cell offspring of *Eed*^*hypo/hypo*^ males. Most notably, *Mad2l1*, which inhibits cell cycle progression, was decreased 11-fold and in heterozygous progeny of *Eed*^*hypo/hypo*^ males compared to progeny of wild-type males, indicating that this gene may regulate the advanced cleavage rate in *Eed*^*hypo/hypo*^ progeny. However, the roles of *Mad2l1* and other cell cycle genes identified here have not been established in preimplantation embryo cleavage and further work is required to ascertain their functional roles in this process.

Interestingly, germline de novo mutations in either *EED* or *EZH2* result in Weaver syndrome, characterised by growth and congenital defects and cognitive deficit in affected humans [62–65]. The maternal/paternal inheritance pattern in Weaver syndrome is poorly understood, although there is some evidence that mutations occur in either the maternal or paternal allele in the germline suggesting that disruption of PRC2 function in either sperm to oocytes may contribute to Weaver syndrome. In this study, partial loss of EED function in the paternal germline was sufficient to mediate significant, though relatively subtle changes in epigenetic and transcriptional regulation in paternal offspring, but not the spectrum of phenotypic characteristics observed in Weaver syndrome patients. Whether greater loss of PRC2 function in male germ cells or in the maternal germline will lead to increased Weaver-like phenotypic changes in mice is yet to be determined. However, loss of EZH2 function in oocytes led to decreased birth weight in mice, rather than increased birth weight typically observed in Weaver syndrome [66].

Surprisingly, despite significant dysregulation of H3K27me3 enrichment on TEs and decreased male fertility, we observed a substantial number of TEs at which H3K27me3 was increased in *Eed* hypomorphic male germ cells. The reason for this is not known, although one possibility is that that silencing of retrotransposable elements is given functional priority in the germline, even when PRC2 activity is compromised. Such a mechanism may reduce the potential detrimental effects of these elements in the germline and the next generation. This may retain individual fitness for the animal, despite the introduction of epigenetic variation due to altered epigenetic control in the germline, such as that observed in this study.

## Conclusions

The current study was designed to determine whether EED regulates epigenetic patterning in the paternal germline that subsequently alters outcomes in offspring. This appears to be the case as both regulation of transcription and preimplantation development were altered in offspring of males with reduced EED function. As existing evidence indicates that EED function is restricted to the establishment of H3K27me3 through PRC2, the simplest interpretation of our data is that PRC2 alters epigenetic patterns in sperm that are manifest in offspring. Reduced PRC2 may have altered the establishment of other epigenetic information to compensate for the change in H3K27me3. This could include changes in RNA content in the sperm that could alter gene expression and embryo development [67, 68]. However, ultimately these changes would occur as a consequence to the original alteration of H3K27me3 due to the reduced function of EED and PRC2. Therefore, this study provides the first functional evidence that the highly conserved histone-modifying complex, PRC2, mediates paternal transmission of inherited effects in mammals. This complements recent evidence that histone modifications play essential roles in regulating inherited disease [60, 69], and emphasises the importance of understanding mechanisms that regulate transmission of epigenetic information through the germline inheritance.

## Methods

### Mice

*Eed* hypomorphic (*Eedhypo/hypo*) mice were generated by inter-crossing *c57bl/6:129T2SvJ Eedl7Rn5-1989SB.Oct4GFP* heterozygous (*Eedwt/hypo*) *mice. Eed*^*l7Rn5-1989SB*^ C57bl6/129 mice were maintained under a light-dark cycle in a temperature and humidity-controlled specifically pathogen-free (SPF) facility with access to food and water ad libitum.

### Embryo collection and staging

Animals were time mated and females were inspected for plugs each morning to ensure successful mating. Embryos were collected at fertilisation, 8.5, 12.5 and 15.5 days after the female was plugged.

Zygote to blastocyst development was monitored as previously described [70]. Briefly, embryos collected at fertilisation were kept warm in G-MOPS medium during transfer to the embryo culture facility before washing twice through 50/50 G1/G2 embryo culture media and transferred into 2 μl drops of G1/G2 media under oil. Embryos were cultured individually in 6% CO_2_, 5% O_2_ and 89% N_2_ for 96 h in an incubator (Sanyo MCO 5) equipped with a Primo Vision (Vitrolife, Sweden) Time Lapse Embryo monitoring system allowing morpho-kinetic analysis of embryo development. Morpho-kinetic development of each embryo was documented using time-lapse photography, with images collected every 10 min for the zygote-blastocyst developmental period. Embryo morphology and cleavage times between zygote to two-cell, two-cell to four-cell, two-cell to eight-cell, and two-cell embryos to blastocyst were documented within Primo Vision and statistically analysed using GraphPad Prism. After culture, embryos were collected and individually snap frozen for genotyping.

To identify differences in transcriptional control in offspring of *Eed*^*hypo/hypo*^ males, E8.5 embryos were produced by *Eed*^*hypo/hypo*^, *Eed*^*hypo/wt*^ and *Eed*^*wt/wt*^ males mated to wild-type females. Embryos were dissected at E8.5, the physical appearance of each embryo was documented and each embryo was photographed before snap freezing in liquid nitrogen. Photographs and notes were later compared to accurately match samples of the same developmental time points and facilitate accurate gene expression analysis and comparison to controls. All E8.5 embryo samples were kept at − 80 °C until RNA extraction. E12.5 and E15.5 embryos were examined on collection to ensure they were consistent with E12.5 and E15.5 developmental stage and gonads were dissected. Gonads were fixed for immunofluorescent analysis, or were dissociated and prepared for FACS purification of germ cells.

### Genotyping

The *Eed*^*1989*^ T > A point mutation was detected in embryos by reverse transcribing RNA using SuperScript® III Reverse Transcriptase Kit (Life Technologies # 18080–051). Samples were PCR amplified using *Eed*-specific primers (forward: 5′- TCACAGGGGGAGATACGGTTATT and reverse: 5′-CTGACAGGAGAAGGTTTGGGTCT) cleaned using ExoSAP-IT (Affymetrix, 78250) and the cDNA subjected to Sanger sequencing at the MHTP Medical Genomics Facility. Resulting sequences were assessed using FinchTV Geospiza software.

### Fertility testing

A controlled breeding experiment was performed to determine the fertility of the male *Eed* hypomorphic mice [71]. *Eed*^*hypo/hypo*^ males were witnessed performing mounting behaviours and successfully produced plugs. The number of pups produced from each female after 1 month was recorded. Each male was housed with two virgin 6-week-old wild-type female mice for 1 month, before replacing the females with another two virgin 6-week-old wild-type females. The number of pups from each female was counted and recorded. If the female was not pregnant after a month, her litter size was counted as 0. Average litter size from four females was calculated for each male and grouped by genotype. Average group litter size was analysed using Bartlett’s *F*-test to compare variances for the three groups and a non-parametric Mann-Whitney test to determine statistical significance. A chi-square test was used to statistically assess differences in the occurrence of successful pregnancies between males grouped by genotype. Fertility was assessed in 13 *Eed*^*hypo/hypo*^ males (*n* = 13) along with age-matched *Eed*^*hypo/wt*^ (*n* = 13) and *Eed*^*wt/wt*^ (*n* = 10) brothers.

### Histology

Testes from *Eed*^*hypo/hypo*^ (*n* = 21), *Eed*^*hypo/wt*^ (*n* = 23) and *Eed*^*wt/wt*^ (*n* = 19) males were processed for histology. Each testis was weighed and the testis capsule nicked, immersed in Bouin’s fixative overnight, washed three times in 70% ethanol (vol/vol), processed into paraffin wax and stained with periodic acid Schiff (PAS) reagent and haematoxylin. Assessment of testis histology was carried out to determine the presence, or absence, of all germ types and their morphological integrity in comparison with wild-type mice of the same age. Initially, one testis was snap frozen and stored at − 80 for sperm count and hormone assessment while the other testis was used for histological analysis. However, after the observation that there was no difference in DSP, all future gonads were fixed for histology assessment.

### Daily sperm production

Frozen testes from *Eed*^*hypo/hypo*^ (*n* = 5), *Eed*^*hypo/wt*^ (*n* = 6) and *Eed*^*wt/wt*^ (*n* = 3) were allowed to thaw at RT, weighed before a fragment was removed, weighed, decapsulated and homogenised in 600 μl of SMT solution. Ten microlitres of homogenate was placed on each side of the haemocytometer. The average number of sperm heads was calculated from counting 80 small squares on both sides of the haemocytometer. Daily sperm counts were calculated as previously reported [72, 73]. Briefly, the volume of homogenate, weight of the sample fragment and total weight of the testis were used to calculate the total number of spermatids per testis. As developing spermatids spend 4.84 days in steps 14–16 during spermatogenesis, the values for the number of spermatids per testis were divided by 4.84 to obtain daily sperm production. Statistical significance was determined using one-way ANOVA with Tukey’s multiple comparison, with *P <* 0.05 considered significant.

### Immunofluorescence

Embryos were harvested at E12.5 and E14.5, sexed based on gonad morphology or via PCR [74]. Foetal gonads were isolated and fixed at RT in PBS containing 4% paraformaldehyde for 20 or 75 min respectively. Gonads were washed three times in PBS and cryoprotected in 30% sucrose in PBS overnight and mounted in optimal cutting temperature (OCT). Cryosections were cut at 8 μm, permeabilised with 1% Triton-X and non-specific staining blocked with 5% BSA. Immunofluorescence staining was carried out as described [75, 76]. EED (R&D Technologies, AF5827, diluted 1/100), EZH2 (Cell Signalling Technology D2C9, diluted 1/400), SUZ12 (Cell Signalling Technology D39F6, diluted 1/100), H3K27me3 (Cell Signalling Technologies, C36B11, diluted 1/400), OCT4 (Santa Cruz sc8628, diluted 1/500), DPPA4 (R&D Systems AF3730, diluted 1/400) and MVH/DDX4 (Cell Signalling Technology #8761, diluted 1/300) primary antibodies were each diluted in PBS containing 1% BSA incubated for 1 h at RT. Donkey anti-goat, sheep or rabbit Alexa-594 (Life Technologies) secondary antibodies were used at 1/500 dilution, while eGFP fluorescence was detected directly in the 488-nm channel. To assess non-specific staining, additional sections were analysed using secondary antibody only controls. Images were obtained using a Nikon® C1 confocal microscope. Images were visually analysed using ImageJ (version: 2.0.0-rc-19/1.49m). All IF experiments were replicated using three to five pairs of gonads per genotype.

### Flow cytometry

Pregnant mothers were injected intraperitoneally (i.p.) with 20 mg/kg 5-ethynyl-2-deoxyuridine (EdU) to facilitate in vivo analysis of gonadal cell proliferation. Flow cytometry was performed as previously described [40, 77, 78]. Dissociated gonadal cells were stained using antibodies specific for SOX9 and AMH allowing identification of Sertoli cells and SOX9 (Millipore AB5535, diluted 1/300) and AMH (Santa Cruz sc-6886, diluted 1/200) staining intensity in the Sertoli cell population. Cell cycle was measured in *Eed*^*wt/wt*^, *Eed*^*wt/hypo*^ and *Eed*^*hypo/hypo*^ samples as previously described [77, 78]. Germ cells were identified using an antibody specific for MVH (R&D Systems, AF2030 diluted 1/100; [77, 78]).

### Fluorescence-activated cell sorting

Fluorescence activated cell sorting was performed essentially as described [42, 75, 76]. Foetal gonads were collected at E15.5 and sexed based on gonadal morphology. Male gonad pairs were dissociated to single cells in 0.25% trypsin containing EDTA. Trypsin activity was blocked by adding 500 μl DMEM containing 10% FBS. The cells were filtered through an 80-μm nylon mesh (BD Biosciences), pelleted and resuspended in 300 μl PBS containing 0.4% BSA for FACS. GFP-positive (germ cells) and GFP-negative (somatic cells) were collected at > 95% purity using a BD Influx Cell Sorter (BD Biosciences). Propidium iodide (200 ng/ml) was added to cell suspensions to monitor cell viability, and only viable cells (> 95%) were collected. Germ cells were pelleted and either fixed for ChIP or snap frozen for RNA. Each gonad pair yielded approximately 20,000 germ cells, and similar proportions of GFP-positive germ cells were isolated from *Eed*^*wt/wt*^, *Eed*^*wt/hypo*^ and *Eed*^*hypo/hypo*^ foetuses (Fig. 1h). We were unable to isolate any *Oct4*GFP-negative germ cells.

### RNA extraction and quality assessment

RNA was extracted from E8.5 embryos using the Genelute Mammalian total RNA miniprep kit (Sigma, RTN70-1KT), DNAse treated using TURBO DNA-free™ Kit (Ambion, AM1907) and purified from the DNAse reaction using Agencourt RNAClean XP (Beckman coulter, A63987). RNA was extracted from pools of eight to ten carefully staged eight-cell embryos, with each pool representing separate litters using Agencourt RNAClean XP (Beckman coulter, A63987) chemistry and the RNA DNAase treated on the beads, before elution, freezing and storage at − 80 °C. Isolation of E15.5 germ cells was performed by FACS purifying approximately 15,000 germ cells from each pair of E15.5 foetal testes. RNA was extracted from the germ cells using the RNeasy Micro Kit (Qiagen) including an on column DNAse step. For all samples isolated from E8.5 embryos, E15.5 germ cells and eight-cell embryos, RNA quantity and quality were measured using a Qubit® RNA HS Assay Kit and the Qubit® 2.0 Fluorometer (Life Technologies, Q32866) and an Agilent 2100 Bioanalyzer (Agilent Technologies, Santa Clara, CA). Only samples with a RIN score above 8 were used for RNA sequencing and microarray analysis.

### Quantitative real-time RT-PCR (qRT-PCR) using Fluidigm biomark

RNA was extracted form 50-200K FACS purified E15.5 testis somatic cells or from whole E12.5 gonads using 600 ml of Trizol in 2Ml heavy phase-lock tubes. DNAsed using Ambion Turbo kit and cDNA synthesised using super script III (Invitrogen) kit. Gene expression was analysed using real-time quantitative polymerase chain reaction with BioMark HD technology (Fluidigm) 96.96 Dynamic Array IFCs (Fluidigm). The geometric CT mean of reference genes Canx, Sdha, and Mapk1 was used to calculate the relative gene expression using the delta-delta CT method. We have shown previously that *Canx*, *Sdha*, and *Mapk1* are expressed at stable levels in E12.5–E15.5 foetal germ cells and somatic cells [75]. Statistical significance was determined using one-way ANOVA with Tukey’s multiple comparison test for *Eed*^*hypo/hypo*^ (E12.5 *n* = 7, E15.5 *n* = 14), *Eed*^*hypo/wt*^ (E12.5 *n =* 7, E15.5 *n* = 12) and *Eed*^*wy/wt*^ (E12.5 *n* = 5, E15.5 *n* = 11). Where variances were unequal, a non-parametric test was used (Mann-Whitney). A *P* value of less than 0.05 was considered significant.

### RNA sequencing

RNA sequencing libraries were constructed from 50 ng, 25 ng or ~ 2 ng of total RNA isolated from E8.5 embryos, E15.5 germ cells or eight-cell embryos, respectively. RNA sequencing libraries were constructed using Nugen Mondrian SPIA Library preparation using Nugen protocol M01335v2 after ribosomal depletion of total RNA using the RiboZero kit (Illumina, RS-122-2201). RNA sequencing was performed on an Illumina HiSeq 1500 instrument with condition sequenced in four biological replicates, derived from ≥ 3 independent sires (for E8.5 and eight-cell embryos) or from four embryos from multiple litters (E15.5 germ cells). Quality filtering and adaptor removal was performed using the Trimmomatic software tool with default trimming parameters [79]. Reads were mapped to the mouse genome, version mm10, using TopHat [80] and genes identified and compared to Ensembl using the RNA-eXpress analysis tool [81]. Replicate quality was assessed by applying multiple dimensional scaling plots, and differential expression analysis was performed using the voom-limma analysis workflow applying empirical Bayes *F*-test [82]. Transcript enrichment was performed based on HOMER-assembled annotations with statistical significance of enrichment assessed by Fisher exact test with Benjamini-Hochberg false detection correction. Pseudogene classification was performed using RetroFinder program (Retroposed Genes V6, UCSC). Data was visualised using the ggplot package within the R analysis environment. Gene set enrichment analysis was performed using MSigDB collections [83] with enrichment assessed by Fisher exact test with Benjamini-Hochberg. Sequence data is available through the European Nucleotide Archive (ENA) accession numbers ERP106776 (E15.5 germ cell data), ERP010195 (8.5-day embryo data) and ERP013725 (eight-cell embryo data).

### Expression microarray analysis

RNA labelling was performed using the one-colour Low RNA Input Quick Amp Amplification Kit (Agilent) and hybridised to the SurePrint G3 Mouse GE 8x60K microarrays according to the manufacturer’s instructions (Agilent). Slides were scanned on an Agilent High-Resolution C DNA Microarray Scanner (G2505B), the features extracted with Feature Extraction 11.0.1.1 and the data analysed to obtain background-subtracted, spatially detrended processed signal intensities. Agilent flagged features were excluded from further analysis. Differential expression was assessed in GeneSpring GX version 12 (Agilent). Percentile shift normalisation was performed, and the probe sets were filtered to exclude the lowest 20% of probes and those not expressed in at least four of the eight (*Eed*^*wt/hypo*^ vs *Eed*^*wt/hypo*^ offspring—array 1) or eight (*Eed*^*wt/wt*^ vs *Eed*^*wt/wt*^ offspring—array 2) samples. Differential expression was reported where twofold change was observed applying a 0.01 FDR after Benjamini-Hochberg multiple testing correction. Data was visualised in a hierarchically clustered heat map within the GeneSpring software package. Raw data is available through the NCBI Gene Expression Omnibus GSE68213 (composed of GSE68212 and GSE68211).

### Chromatin immunoprecipitation (ChIP)

ChIP was performed on approximately 20,000 FACS purified germ cells from individual *Eed*^*wt/wt*^ (*n* = 4) and *Eed*^*hypo/hypo*^ (*n =* 4) embryos using low input ChIP [84, 85] with minor alterations. Briefly, cells were cross-linked in 1% formaldehyde/PBS for 5 min before adding glycine/PBS at a final concentration of 125 mM. Cells were pelleted and washed in PBS and stored at − 80 °C. Dynabeads were washed in RIPA buffer and bound to 2.4 μg of H3K27me3 rabbit polyclonal (Millipore cat# 07–449, 1 mg/ml) or IgG rabbit CST (cat#3900S, 2.5 mg/ml) by incubating for a minimum of 2 h at 4 °C at 40 rpm.

Cells were thawed on ice and lysed in 13 μl of lysis buffer for 3 min, vortexed for two 5-s bursts at room temperature and 117 μl modified RIPA buffer containing Tris-HCl pH 7.5 EGTA, Triton X-100 0.1% SDS. Sonication conditions were pre-optimised by assessing germ cell-derived sonicated chromatin samples on a gel and via bioanalyser High Sensitivity DNA Analysis Kits (Agilent Technologies) to ensure that the majority of chromatin fragments yielded were within a 300–100-bp range. Germ cell chromatin was sonicated for 10 min at peak power 105, duty factor 2.0, cycles/burst 200 using a Covaris s220 instrument. Sample was recovered and 5 M NaCl added to a final concentration of 140 mM (equivalent to RIPA buffer). A 10 μl aliquot of each sample was removed for an input control and the remaining chromatin solution immediately transferred to the antibody-bead complexes. The chromatin was incubated with the antibody-bead suspension at 4 °C rotating at 40 rpm for 2 h before washing in cold RIPA buffer and resuspending TE. To reverse crosslink, chromatin-bead suspensions and input controls were resuspended in elution buffer containing freshly added SDS and Proteinase K and incubated for 2 h at 68 °C 13,000 rpm. DNA was purified for sequencing using Agencourt Ampure XP beads (Beckman Coulter).

H3K27me3 rabbit polyclonal (Millipore cat# 07-449, 1 mg/ml) antibody specificity was validated using Active Motif Modified Histone Array analysis (Catalogue number 13001) according to the array manufacturer’s protocol, with the exception that dilution of the primary antibody (1/2000) was as recommended by Millipore for western blotting. Mouse anti Rabbit HRP secondary antibody was used at the recommended dilution (1/2500). Imaging of the array was performed using incremental accumulating exposures to ensure data was collected in the linear detection range. Data analysis was performed using Array Analyse Software (Active Motif) and the specificity factor determined for the antibody with reference to 384 histone peptide variants on the array (Additional file 1: Figure S7).

### ChIP sequencing and analysis

ChIP-Seq Libraries were prepared from using the Ovation Ultralow System V2 using Nugen protocol M01379v1. Each library was quantitated using a Qubit instrument and the DNA size profile determined using an Agilent Bioanalyzer. Libraries were finally quantitated by qPCR, pooled in an equimolar ratio and all eight libraries run on one lane of a HiSeq1500 sequencer to obtain 50-bp single end reads. Raw reads were bioinformatically separated into individual libraries and trimmed using the trimmomatic tool [79] before mapping. Sequences were mapped to the mouse genome (mm10) using bowtie2, resulting in over 95% quality mappable reads. PCR duplicates were marked in the mapping files (BAM format) for filtering in further steps, ensuring use of unique reads during peak identification. Peak identification and differential peak identification was performed using HOMER (http://homer.salk.edu/homer/).

Differential peaks were annotated using HOMER, allowing all peaks to be assigned to specific sequence classes. Genomic sequence classes outside repeats were analysed for over-, under- or expected representation in sequences differentially enriched for H3K27me3 in *Eed*^*hypo/hypo*^ germ cells compared to *Eed*^*wt/wt*^ germ cells. Initially, the expected number of peaks was calculated by determining the proportion of the genome covered by each sequence class (e.g. for intergenic sequences 813,929,088 bp) divided by the total annotated genome size (i.e. 813,929,088/2,631,564,759 = 30.93%). The number of peaks in each sequence class (e.g. for intergenic sequences with increased H3K27me3 in *Eed*^*hypo/hypo*^ germ cells: 380) was divided by the total number of peaks for which increased H3K27me3 was detected (e.g. peaks with increased H3K27me3: 1158). Over-, under- or expected representation was determined by calculating whether the actual percentage represented in the differentially enriched sequences (e.g. for intergenic sequences with increased H3K27me3: 380/1158 = 41.17%) significantly differed from the expected values (for intergenic sequences = 30.93%) based on genome-wide representation for each sequence class using a chi-square test to compare expected and observed values. ChIPseq data is available through the NCBI Gene Expression Omnibus accession number GSE110529.

Representation of repeat sequences was determined by determining the numbers of repeat sequences annotated in each sequence category compared to the total number of repeats in the genome to provide the expected representation of repeats in each repeat category annotated in UCSC repeat masker. A hypergeometric analysis was performed to determine representation of each repeat category in all H3K27me3 peaks identified in *Eed*^*wt/wt*^ and *Eed*^*hypo/hypo*^ germ cells and in peaks that had altered H3K27me3 in *Eed*^*wt/wt*^ compared to *Eed*^*hypo/hypo*^ germ cells, and vice versa.

ChIP data from the current study was compared with that reported by Mu et al. [32] and Ng et al. [29] in which H3K27me3 peaks were identified using MACS. Polycomb target genes from Boyer et al. [86] were transposed to the current mm10 nomenclature and H3K27me3 peaks mapped in the genic and upstream regions of these Polycomb target genes in the current study and studies from Mu et al. [32] and Ng et al. [29].

### Phenotypic analyses of offspring

Two *Eed* hypomorphic males from each genotype (*Eed*^*hypo/hypo*^ and *Eed*^*hypo/wt)*^ were each bred with three C57Bl/6J females, to obtain seven and six litters, respectively. Offspring from *Eed*^*hypo/hypo*^ (*n* = 56) and *Eed*^*hypo/wt*^ (*n* = 48) fathers were weighed at post-natal day 5; crown-rump and nose-rump measurements were obtained. Offspring from *Eed*^*hypo/wt*^ fathers were genotyped via Transnetyx to analyse wild-type and heterozygous groups. All litters analysed consisted of six to ten pups. One-way ANOVA plus Tukey’s post hoc multiple comparisons test was used to statistically analyse the data. A comprehensive phenotypic analysis was also performed using the Australian Phenomics Network Histopathology and Organ Pathology Service. Neonates were weighed and crown-rump and nose-rump lengths collected. The neonates were decapitated, injected with Bouin’s fixative into the thorax and abdomen as well as immersion fixation for 48 h, washed and stored in 70% EtOH. Analysis included evaluation of all thoracic and abdominal organs, skeletal tissue, nasal/oral region, brain, eyes and auditory/vestibular apparatus. Reports were generated for each neonate, including detailed histopathological and neuropathology descriptions.

## Additional files

Additional file 1: **Figure S1.** Survival and fertility of *Eed*^*hypo/hypo*^ animals. **Figure S2.** Foetal somatic and germ cells reached expected developmental milestones and expressed endogenous germ cell markers in *Eed*^*hypo/hypo*^ males. **Figure S3.** H3K27me3 was enriched at known PRC2 target genes in *Eed*^*hypo/hypo*^ compared to *Eed*^*wt/wt*^ germ cells. **Figure S4.** Read counts and technical consistency between RNAseq samples generated from (A–B) E15.5 *Eed*^*wt/wt*^ and *Eed*^*hypo/hypo*^ male germ cells and (C) D8-cell offspring from *Eed*^*wt/wt*^ and *Eed*^*hypo/hypo*^ males. **Figure S5.** Transcriptional analyses of E8.5 offspring produced by *Eed*^*hypo/hypo*^, *Eed*^*hypo/wt*^ and *Eed*^*wt/wt*^ male mice mated to wild-type females. **Figure S6.** Neonatal weight and size are not different in offspring of *Eed*^*hypo/wt*^ *Eed*^*hypo/hypo*^ and males. **Figure S7.** Active Motif Modified Histone Array analysis of H3K27me3 ChIP antibody. (PDF 15192 kb)

Additional file 2: **Table S1.** *Eed*^*wt/wt*^ germ cell H3K27me3 ChIP-seq peaks. (XLSX 6770 kb)

Additional file 3: **Table S2.** *Eed*^*hypo/hypo*^ germ cell H3K27me3 ChIP-seq peaks. (XLSX 8636 kb)

Additional file 4: **Table S3.** Comparison of known PRC target genes in ES cells with H3K27me3 peaks in foetal germ cells, ES cells and spermatocytes. (XLSX 1161 kb)

Additional file 5: **Table S4.** Relative representation of annotated repeat categories in H3K27me3 peaks in *Eed*^*wt/wt*^ and *Eed*^*hypo/hypo*^ germ cells. (XLSX 1124 kb)

Additional file 6: **Table S5.** ChIP-seq data from E15.5 male *Eed*^*hypo/hypo*^ compared to *Eed*^*wt/wt*^ germ cells: Peaks with decreased H3K27me3 in *Eed*^*hypo/hypo*^ germ cells. (XLSX 70 kb)

Additional file 7: **Table S6.** ChIP-seq data from E15.5 male *Eed*^*hypo/hypo*^ compared to *Eed*^*wt/wt*^ germ cells: Peaks with increased H3K27me3 in *Eed*^*hypo/hypo*^ germ cells. (XLSX 83 kb)

Additional file 8: **Table S7.** ChIP-seq data showing regions with decreased H3K27me3 in *Eed*^*hypo/hypo*^ compared *Eed*^*wt/wt*^ to germ cells identified using EdgeR. (XLSX 11 kb)

Additional file 9: **Table S8.** RNA-seq and expression microarray data showing genes differentially expressed in heterozygous E8.5 day embryos sired by *Eed*^*hypo/hypo*^ fathers compared to E8.5-day embryos sired by *Eed*^*hypo/wt*^ fathers. (XLSX 22 kb)

Additional file 10: **Table S9.** RNA-seq data showing genes significantly downregulated in eight-cell embryos sired by *Eed*^*hypo/hypo*^ fathers. (XLSX 20 kb)

Additional file 11: **Table S10.** RNA-seq data showing genes significantly upregulated in eight-cell embryos sired by *Eed*^*hypo/hypo*^ fathers. (XLSX 23 kb)

## Abbreviations

AMH: Anti-Mullerian hormone
ChIP-seq: Chromatin immunoprecipitation followed by genome wide sequencing
DPPA4: Developmental Pluripotency Associated gene 4
DSP: Daily sperm count
E: Embryonic day
EED: Embryonic ectoderm development
eGFP: Enhanced Green Fluorescent protein
EZH1: Enhancer of Zeste 1
EZH2: Enhancer of Zeste 2
FACS: Fluorescence-activated cell sorting
H3K27me3: Trimethylated lysine 27 on histone 3
lincRNA: Long non-coding RNA
LINE: Long Interspersed Nuclear Element
LTR: Long Terminal Repeat
Mad2l1: Mitotic Arrest Deficient 2 Like 1
Mcm3: Minichromosome Maintenance Complex Component 3
MVH: Mouse Vasa Homologue (also known as DDX4)
*Nsd*: No significant difference
OCT4: Octamer Binding Transcription Factor 4 (also known as *Pou5f1*)
Pkmyt1: Protein Kinase Membrane Associated Tyrosine/Threonine 1
PND: Post-natal day
PRC2: Polycomb Repressive Complex 2
RNA-seq: Genome-wide RNA sequencing
Sdha: Succinate Dehydrogenase Complex, subunit A
SINE: Short Interspersed Nuclear Element
SOX9: SRY Box Containing Gene 9
SUZ12: Suppressor of Zeste 12
TEs: Transposable elements
