# Supplementary material for: Reduced PRC2 function alters male germline epigenetic programming and paternal inheritance
Source: BMC Biol. 2018 Sep 20;16:104. doi: 10.1186/s12915-018-0569-5 (PMC6149058; doi:10.1186/s12915-018-0569-5)
Supplement: Supplementary file 1 — Figure S1. Survival and fertility of Eedhypo/hypo animals. Figure S2. Foetal somatic and germ cells reached expected developmental milestones and expressed endogenous germ cell markers in Eedhypo/hypo males. Figure S3. H3K27me3 was enriched at known PRC2 target genes in Eedhypo/hypo compared to Eedwt/wt germ cells. Figure S4. Read counts and technical consistency between RNAseq samples generated from (A–B) E15.5 Eedwt/wt and Eedhypo/hypo male germ cells and (C) D8-cell offspring from Eedwt/wt and Eedhypo/hypo males. Figure S5. Transcriptional analyses of E8.5 offspring produced by Eedhypo/hypo, Eedhypo/wt and Eedwt/wt male mice mated to wild-type females. Figure S6. Neonatal weight and size are not different in offspring of Eedhypo/wt Eedhypo/hypo and males. Figure S7. Active Motif Modified Histone Array analysis of H3K27me3 ChIP antibody. (PDF 15192 kb) [file 12915_2018_569_MOESM1_ESM.pdf]

Supplementary Figures for:

Reduced PRC2 function alters male germline epigenetic programming and paternal inheritance.

Jessica M Stringer[1,2], Samuel C Forster[3,4,5] Zhipeng Qu[6], Lexie Prokopuk[1,5], Moira K O'Bryan[7], David K Gardner[8], Stefan J White[9], David Adelson[6] and Patrick S Western[1,5#]

[1]Centre for Reproductive Health, Hudson Institute of Medical Research, Clayton, Victoria, Australia, 3168.

[2]Ovarian Biology Laboratory, Biomedicine Discovery Institute, Department of Anatomy and Developmental Biology, Monash University, Melbourne, Australia, 3168.

[3]Host-Microbiota Interactions Laboratory, Wellcome Trust Sanger Institute, Hinxton, UK, CB10 1SA.

[4]Centre for Innate Immunity and Infectious Diseases, Hudson Institute of Medical Research, Clayton, Victoria, Australia, 3168.

[5]Molecular and Translational Science, Monash University, Clayton, Victoria, Australia, 3168.

[6]Bioinformatics and Computational Genetics, School of Biological Sciences, The University of Adelaide, South Australia, Australia, 5005.

[7]School of Biological Sciences, Monash University, Clayton, Victoria, Australia, 3168.

[8]School of BioSciences, University of Melbourne, Parkville, Australia.

[9]Leiden Genome Technology Centre, Department of Human Genetics, Leiden University Medical Center, Leiden, the Netherlands

A

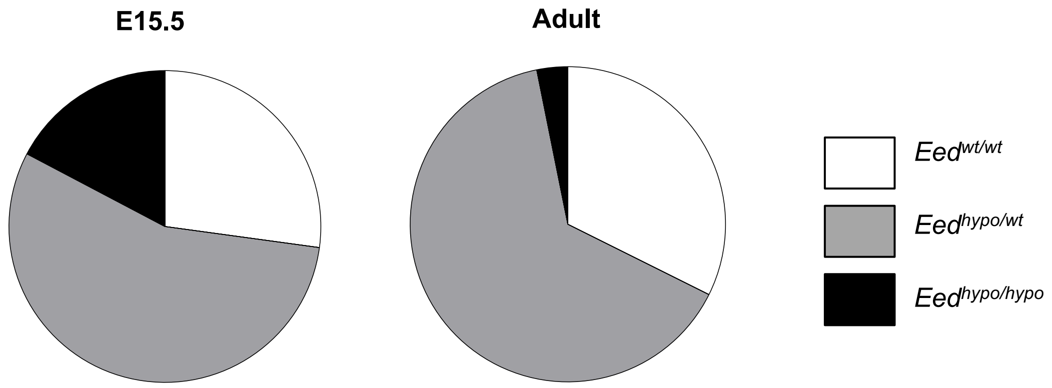

Total = 162

Chi-squared P<0.08

Total = 411

Chi-squared P<0.001

B

Litter size frequency grouped by male genotype.

**Paternal genotype**	**not pregnant (no litter)**	**1-7**	**8-13**	**Total**
Eed$^{wt/wt}$ n=10	0 (0)	21.1 (8)	78.9 (30)	100 (38)
Eed$^{hypo/wt}$ n=13	0 (0)	8.3 (4)	91.7 (44)	100 (48)
Eed$^{hypo/hypo}$ n=13	25.5 (12)	10.6 (5)	63.8 (30)	100 (47)
Total	9.0 (12)	12.8 (17)	78.2 (104)	100 (133)
Chi-Square	27.26**	P=1.8E-05		

C

Litter size related to male age

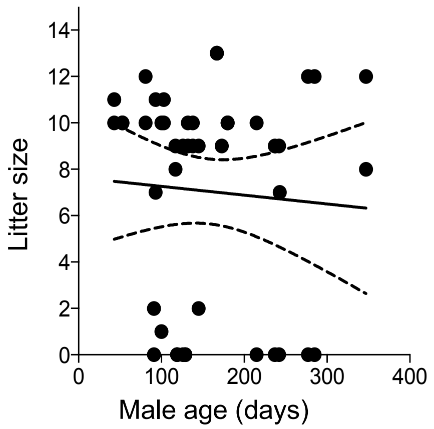

Supplementary Figure 1: Survival and fertility of *Eed*$^{hypo/hypo}$ animals. (A) Percentage of live *Eed*$^{wt/wt}$, *Eed*$^{hypo/wt}$, *Eed*$^{hypo/hypo}$ animals collected at E15.5 (n=162) and >PND2 (n=411). Data analysed using a *Chi*-squared test with $P<0.05$ regarded as significant. **(B)** *Chi*-square analysis of litter size frequency, grouped by male genotype. Total number of litters in parentheses. **(C)** Litter size related to male age for all males involved in the study (P=0.6209; n=34, 45, 46 for *Eed*$^{wt/wt}$, *Eed*$^{hypo/wt}$, *Eed*$^{hypo/hypo}$).

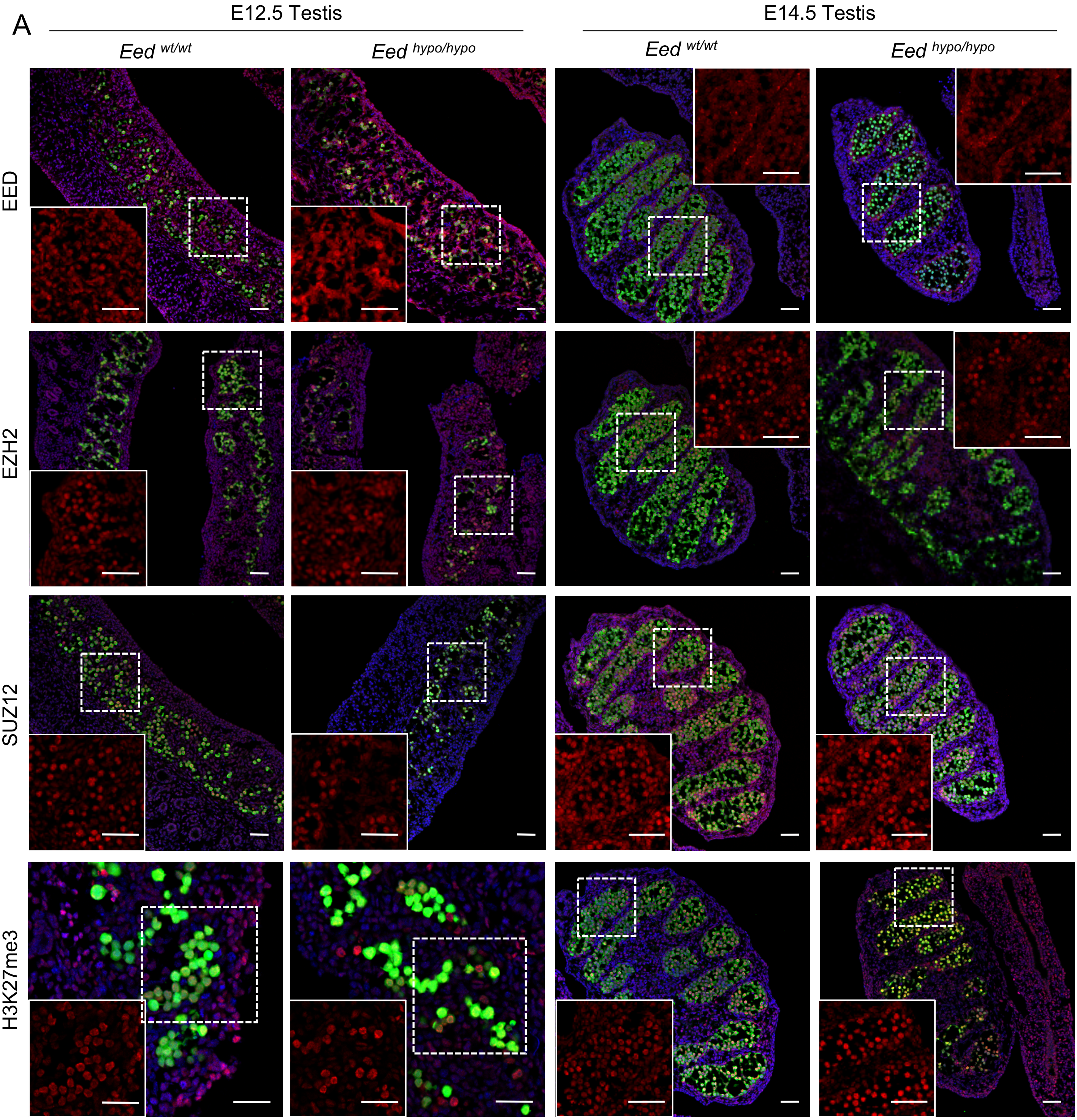

Stringer et al Supp Fig 2

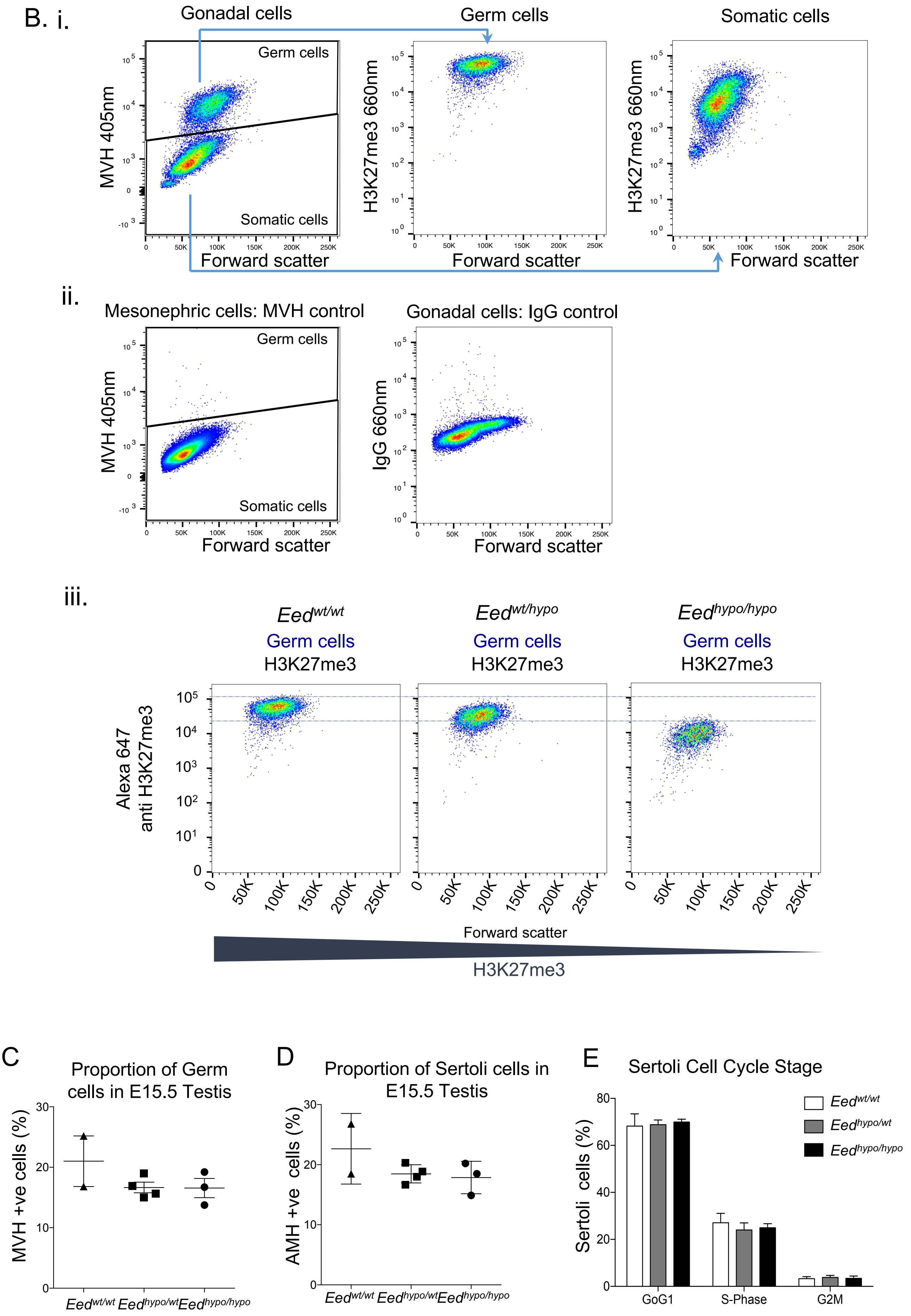

Stringer et al Supp Fig 2

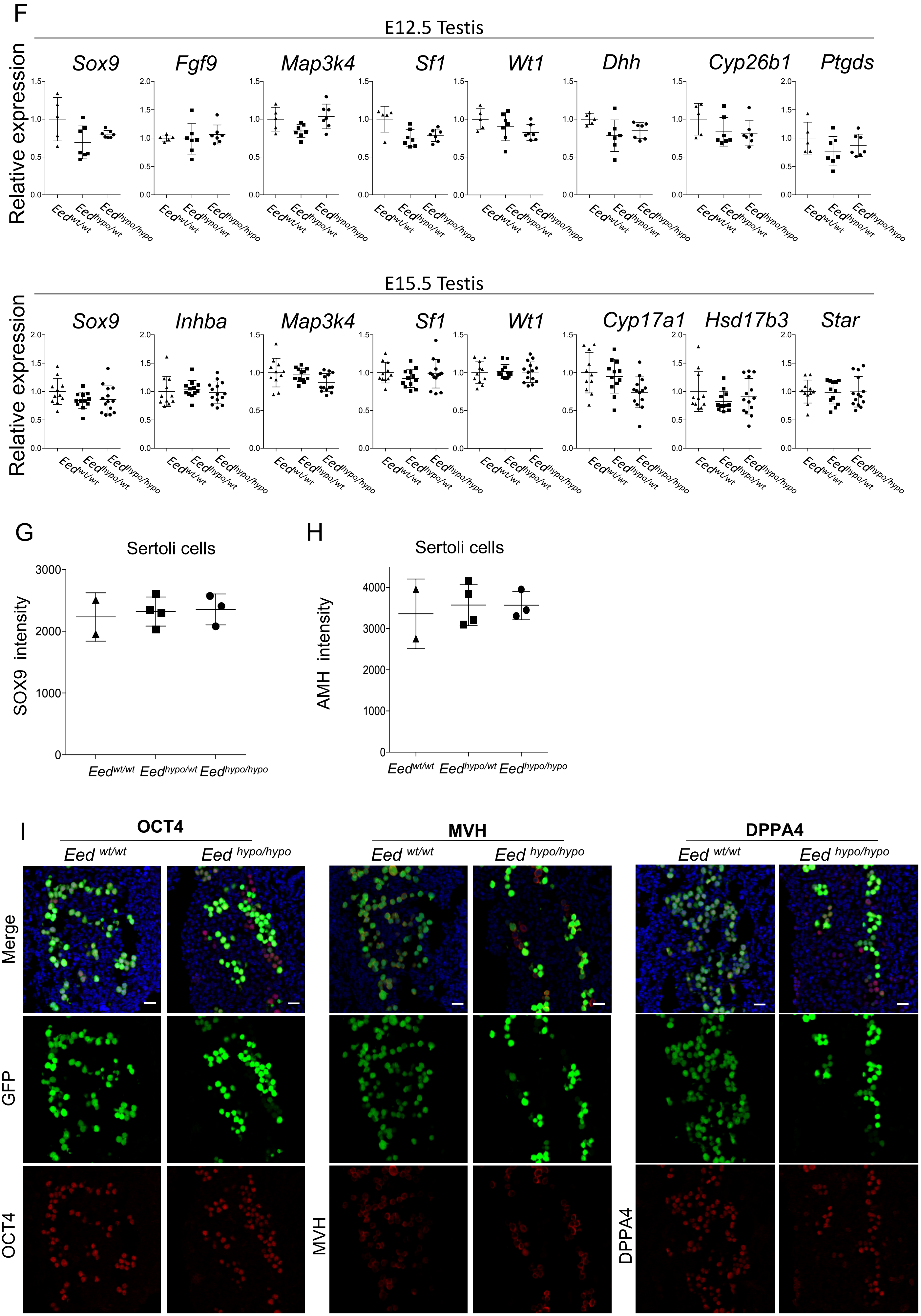
F
E12.5 Testis
Relative expression
Sox9
Fgf9
Map3k4
Sf1
Wt1
Dhh
Cyp26b1
Ptgds
Eed wt/wt
Eed hypo/wt
Eed hypo/hypo
E15.5 Testis
Relative expression
Sox9
Inhba
Map3k4
Sf1
Wt1
Cyp17a1
Hsd17b3
Star
G
Sertoli cells
SOX9 intensity
H
Sertoli cells
AMH intensity
I
OCT4
MVH
DPPA4
Eed wt/wt
Eed hypo/hypo
Merge
GFP
OCT4
MVH
DPPA4

Supplementary Figure 2: Fetal somatic and germ cells reached expected developmental milestones and expressed endogenous germ cell markers in *Eed*$^{hypo/hypo}$ males. **(A)** Representative images of E12.5 and E14.5 testes immunofluorescence analysis of EED, EZH2 and SUZ12 and H3K27me. Germ cells are marked by *Oct4*GFP and/or MVH in green (488nm) EED, EZH2, SUZ12 and H3K27me3 antibodies are shown in Red (594nm) detected using Alexa-flour 594 secondary antibodies, DAPI (blue) marks DNA (n=3-6 per age per genotype). *Eed*$^{hypo/wt}$ images (not shown) looked the same as wild-type. Scale is 50µm. **(B)** Flow cytometric analysis of H3K27me3 levels in E15.5 *Eed*$^{wt/wt}$ (n=5), *Eed*$^{hypo/wt}$ (n=7) and *Eed*$^{hypo/hypo}$ (n=2) testes. **i.** Gating of MVH positive germ cells and MVH negative somatic cells (right scatter plot) **ii-iii.** H3K27me3 staining in germ (ii) and somatic cells (iii). **iv.** Negative controls: mesonephric cells stained using an MVH antibody, and E15.5 gonadla cells stained with an isotype IgG control antibody. **v.** Examples of H3K27me3 staining intensities in germ cells from *Eed*$^{wt/wt}$, *Eed*$^{hypo/wt}$ and *Eed*$^{hypo/hypo}$ testes. **(C-E)** Flow cytometric analysis of the percentage of Sertoli (C) and germ cells (D) and the proliferation of Sertoli cells in E15.5 gonads (E). **(F)** qRT-PCR expression analysis of key genes involved in Sertoli, germ and steroidogenic cell lineage development relative to the average expression of three housekeeping genes (*Canx*, *Sdha*, and *Mapk1*). Data are mean ± SEM, One-way ANOVA; No significant differences. **(G-H)** Flow cytometric analysis of SOX9 (B) and AMH (C) antibody fluorescence intensity as representative of protein levels in Sertoli cells of E15.5. For flow cytometric analyses in B-D and F-G *Eed*$^{wt/wt}$ (n=2) *Eed*$^{hypo/wt}$ (n=4) and *Eed*$^{hypo/hypo}$ (n=3) testes, Data are mean ± SEM, No significant differences. **(I)** Representative images of immunofluorescence analysis of OCT4, DPPA4 and MVH in E12.5 *Eed*$^{wt/wt}$ and *Eed*$^{hypo/hypo}$ testes. Germ cells are marked by Oct4GFP in green (488nm), OCT4, MVH and DPPA4 antibodies are shown in Red (594nm) detected using Alexa-flour 594 secondary antibodies, DAPI (blue) marks DNA (n=4-5 per genotype). *Eed*$^{hypo/wt}$ images (not shown) were similar to wild-type. Scale is 25µM.

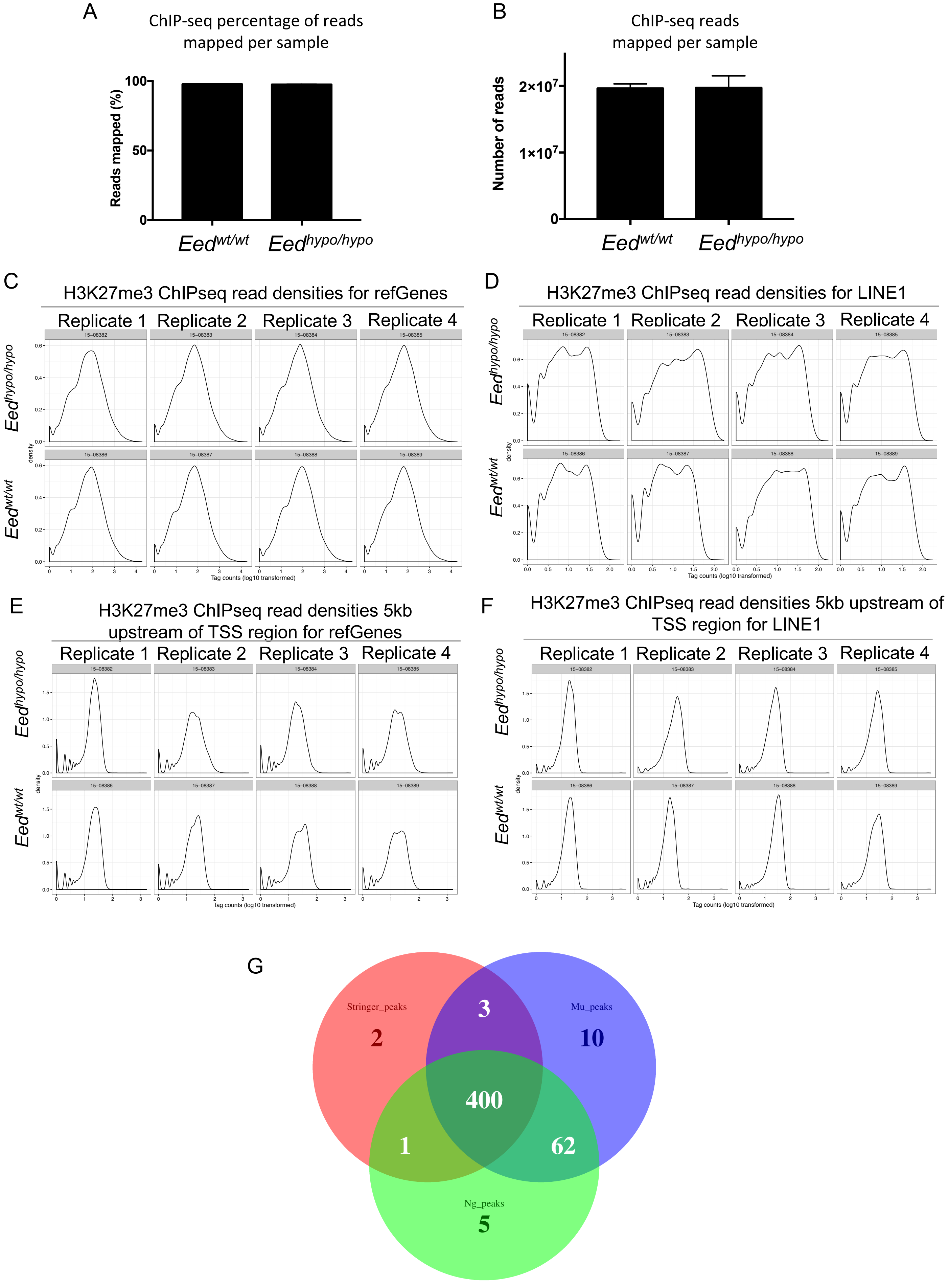

Stringer Supp Fig3

H

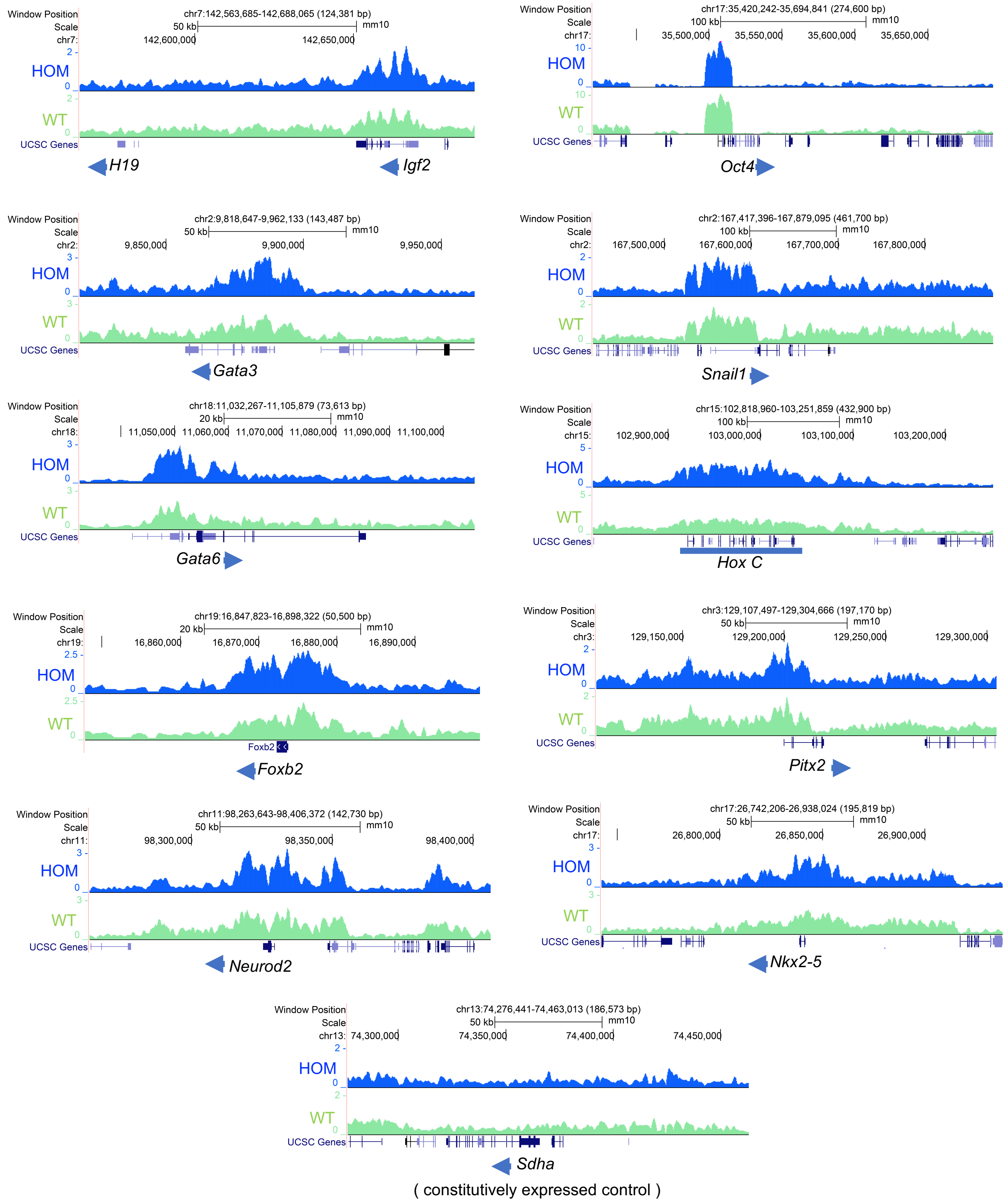

I

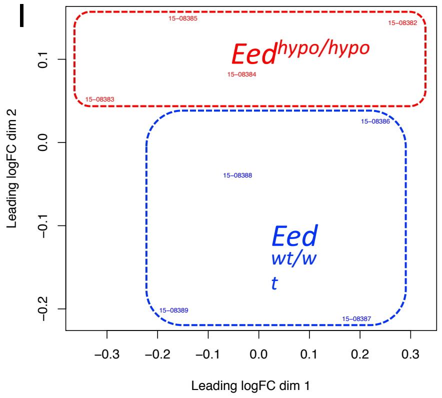

J

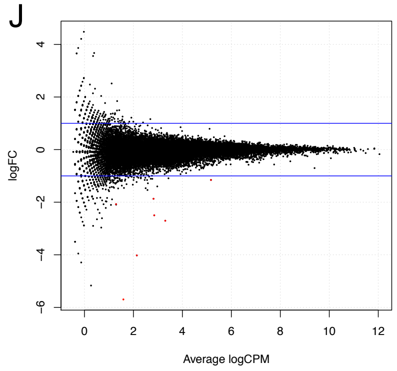

Stringer Supp Fig3

Supplementary Figure 3: H3K27me3 was enriched at known PRC2 target genes in *Eed*$^{hypo/hypo}$ compared to *Eed*$^{wt/wt}$ germ cells. (A) Percentage of ChIP-seq reads from E15.5 *Eed*$^{wt/wt}$ (n=4) and *Eed*$^{hypo/hypo}$ (n=4) germ cells mapped to the genome. Data are mean ± SEM, two tailed t-test; No significant differences. **(B)** Number of ChIP-seq reads from E15.5 *Eed*$^{wt/wt}$ (n=4) and *Eed*$^{hypo/hypo}$ (n=4) germ cells mapped to the genome. Data are mean ± SEM, One-way ANOVA; No significant differences. **(C-F)** H3K27me3 read densities in four replicates of E15.5 fetal male germ cells from *Eed*$^{wt/wt}$ and *Eed*$^{hypo/hypo}$ mice for **(C)** refGenes **(D)** LINE1 **(E)** 5kb upstream of the TSS for refGenes **(F)** 5kb upstream of the TSS for LINE1 **(G)** H3K27me3 enrichment in 491 cross-validated Polycomb target genes identified by Boyer *et al* 2006 [50] intersected with gene specific H3K27me3 enrichment observed in E15.5 fetal male germ cells in this study, E13.5 fetal male germ cells (Ng *et al* 2013: [21]) and spermatocytes (Mu *et al* 2017: [22]). TSS region defined as 2.5kb upstream flanking and 2.5kb downstream region from the TSS. **(H)** E15.5 *Eed*$^{wt/wt}$ and *Eed*$^{hypo/hypo}$ germ cell Chip-seq UCSC tracks showing H3K27me3 enrichment for known PRC2 targets, the *H19-IgfII* imprinted locus and the ubiquitously expressed gene *Sdha,* which is not enriched for H3K27me3. **(I)** PCA plot the 500 most variable loci in eight samples of E15.5 *Eed*$^{wt/wt}$ (n=4) and *Eed*$^{hypo/hypo}$ (n=4) germ cells. **(J)** Maplot analysis of the ChIP data from E15.5 *Eed*$^{wt/wt}$ (n=4) and *Eed*$^{hypo/hypo}$ (n=4) germ cells showing average read counts vs log fold change.

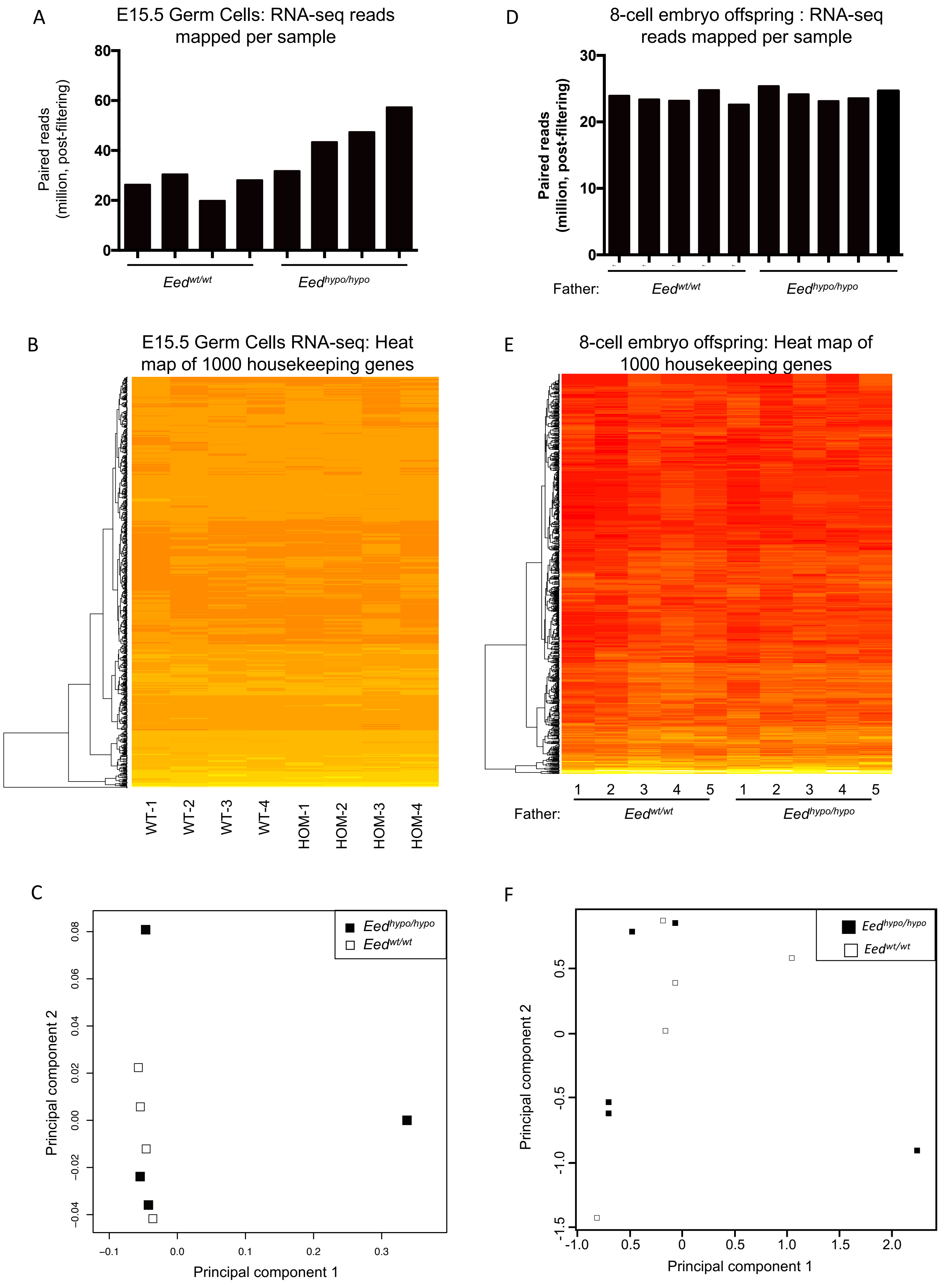

Stringer Supp Fig4

Supplementary Figure 4: Read counts and technical consistency between RNA-seq samples generated from (A-B) E15.5 *Eed* $^{wt/wt}$ and *Eed* $^{hypo/hypo}$ male germ cells and (C) D8-cell offspring from *Eed* $^{wt/wt}$ and *Eed* $^{hypo/hypo}$ males . (A) Number of RNA-seq reads mapped to the genome from four biological replicates of E15.5 *Eed*$^{wt/wt}$ and *Eed*$^{hypo/hypo}$ germ cells. (**B**). Heat map of 1000 genes that were not differentially expressed between *Eed*$^{wt/wt}$ (WT) and *Eed*$^{hypo/hypo}$ (HOM) germ cells revealing consistency between the four biological replicates for each genotype. **(C)** Principal component analysis of RNAseq data from E15.5 germ cells isolated from *Eed*$^{hypo/hypo}$ mice (black squares) and *Eed*$^{wt/wt}$ mice (white squares). Greater variation was observed between the *Eed*$^{hypo/hypo}$ samples than between the *Eed*$^{wt/wt}$ samples. **(D)** Number of RNA-seq reads mapped to the genome from five biological replicates of pooled 8-cell embryo offspring produced by five *Eed*$^{wt/wt}$ and five *Eed*$^{hypo/hypo}$ males. **(E)** Heat map of 1000 genes that were not differentially expressed between 8-cell embryos produced by five *Eed*$^{wt/wt}$ and five *Eed*$^{hypo/hypo}$ males revealing consistency between the five biological replicate pools of 8-cell embryos from each sire genotype. **(F)** Principal component analysis illustrating global gene expression profile of 8-cell embryos produced by *Eed*$^{wt/wt}$ (white) and *Eed*$^{hypo/hypo}$ (black) males (n=5 pools of ~10 embryos/sample).

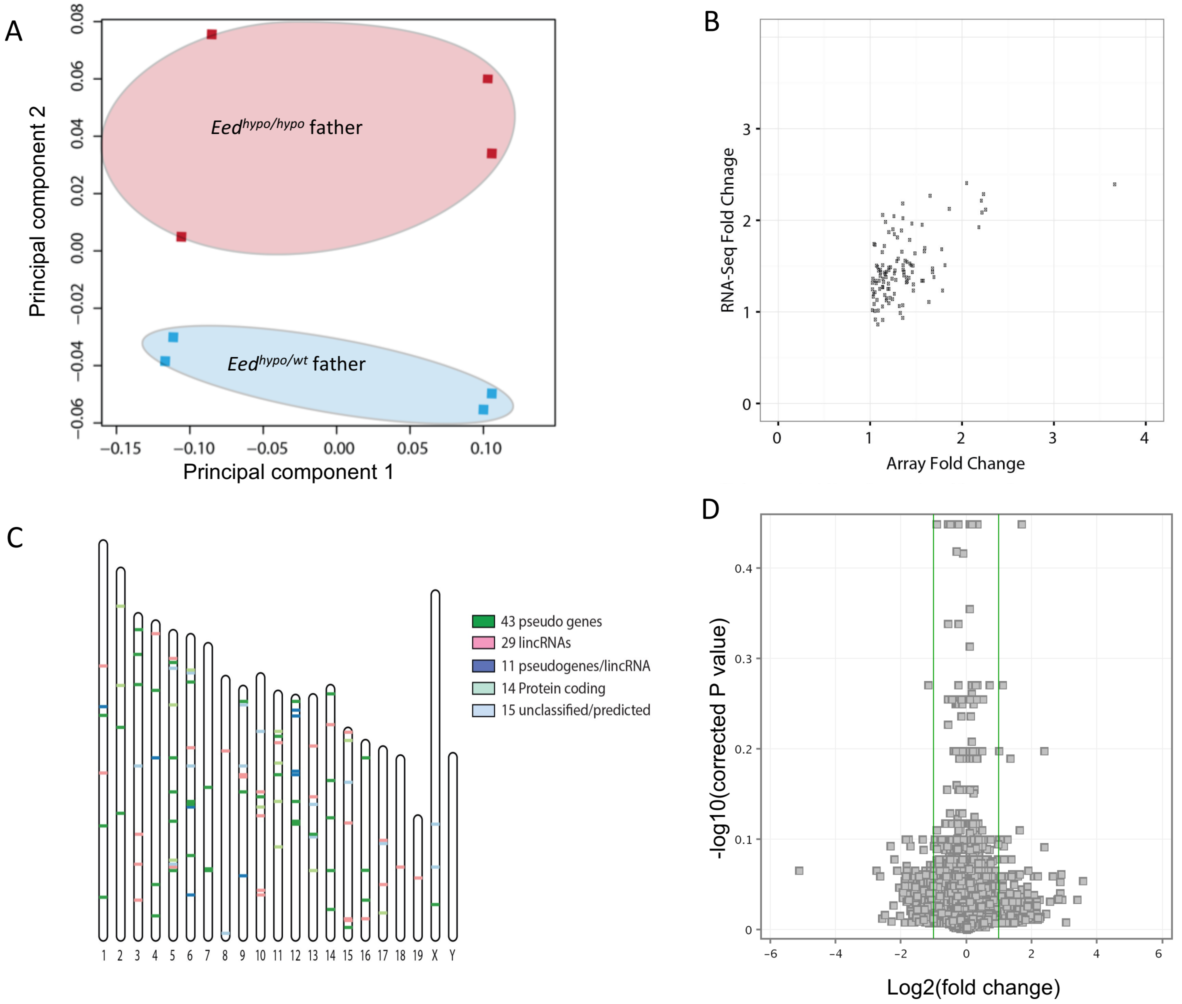

Stringer Supp Fig5

Supplementary Figure 5: Transcriptional analyses of E8.5 offspring produced by *Eed*$^{hypo/hypo}$, *Eed*$^{hypo/wt}$ and *Eed*$^{wt/wt}$ male mice mated to wild type females. (A) Principal component analysis of RNA-Sequencing, illustrating the separation of samples from heterozygous father (Blue) and homozygous father (Red). **(B-C)** RNA-seq and expression microarray analyses of heterozygous E8.5 offspring produced by *Eed*$^{hypo/wt}$ and *Eed*$^{hypo/hypo}$ male mice mated to wild type females. **(B)** Dot-plot (log_2 scale) of differentially expressed transcripts identified using RNA-seq compared to microarray (P<0.01; Benjamini-Hochberg false detection correction). Correlation between RNA-seq and microarray lists was high (R^2=0.87). **(C)** Ideogram showing chromosomal locations functional classification of differentially regulated genes detected using RNA-seq and microarray. Four offspring were analysed from three different fathers for each genotype using independent RNA-seq and expression microarray approaches. Three different *Eed* littermate pairs (i.e. *Eed*$^{hypo/hypo}$ and *Eed*$^{hypo/wt}$ brothers) were used to generate progeny for each genotype. **(D)** Volcano plot showing a comparison of array data from *Eed*$^{wt/wt}$ offspring generated from *Eed*$^{wt/wt}$ and *Eed*$^{hypo/wt}$ males (n=4, for each genotype) mated to c57bl/6J wild type females (n=2 for each male). No differentially expressed transcripts were identified with a cut-off of >2-fold change and P<0.01 with Benjamini-Hochberg false detection correction.

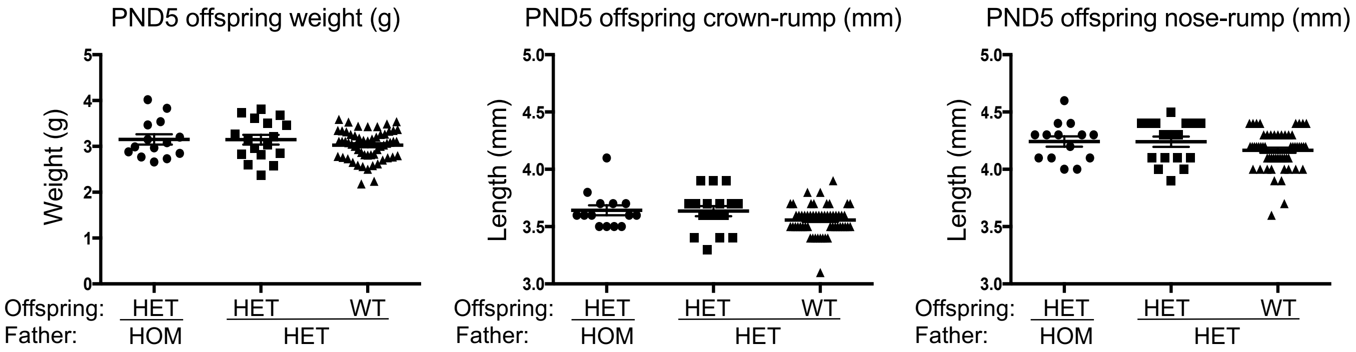

Stringer Supp Fig6

Supplementary Figure 6: Neonatal weight and size are not different in offspring of *Eed*$^{hypo/wt}$ *Eed*$^{hypo/hypo}$ and males. Weight, crown-rump and nose-rump lengths of post-natal day (PND) 5 *Eed*$^{wt/wt}$ (WT), *Eed*$^{hypo/wt}$ (HET) and *Eed*$^{hypo/wt}$ (HOM) offspring generated from *Eed*$^{hypo/wt}$ and *Eed*$^{hypo/hypo}$ males (n=2, for each genotype) mated to c57bl/6J wild type females (n=2 for each male). Pups from 6-7 litters for each genotype (n= 56 and 48; 7-9 pups/litter with one litter of 6 and one litter of 10) were not significantly different in weight, crown-rump or nose-rump lengths. Data are mean ± SEM, One-way ANOVA; No significant differences.

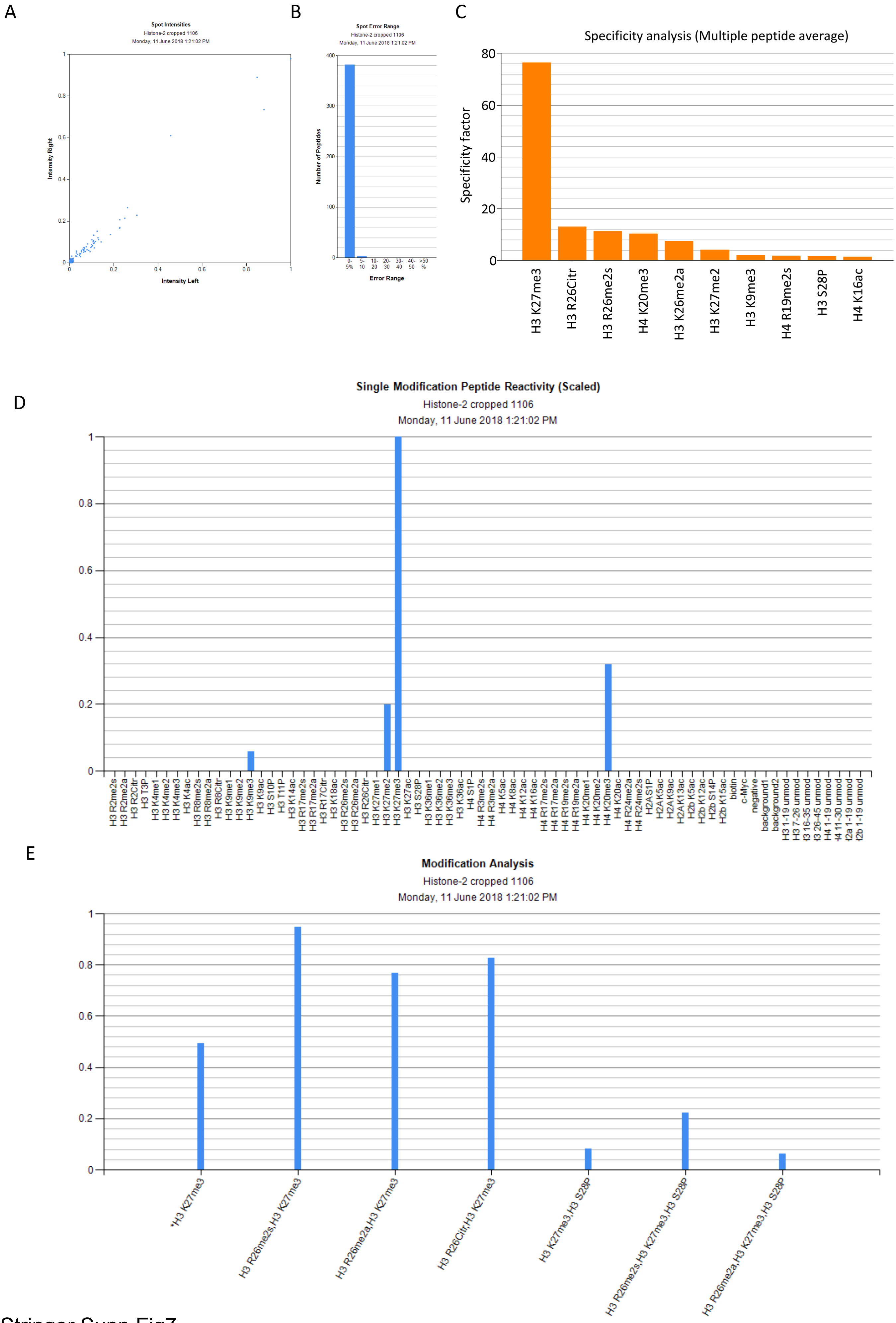

Stringer Supp Fig7

Supplementary Figure 7: Active Motif Modified Histone Array analysis of H3K27me3 ChIP antibody. (A) Comparison of the intensities of each duplicate spot. (B) Array spot distribution errors of the intensities between duplicate spots, normalized to the maximum intensity. (C) Specificity analysis. Top 10 modifications with the greatest specificity factors using multiple peptide average of all spots containing the mark divided by the average intensity of all the spots not containing the mark. (D) Antibody reactivity. Intensity values for all single modified peptides normalized to H3K27me3. (E) Modification analysis. Intensity for all spots containing H3K27me3.
